# The Combination of Δ^9^-Tetrahydrocannabinol and Cannabidiol Suppresses Mitochondrial Respiration of Human Glioblastoma Cells via Downregulation of Specific Respiratory Chain Proteins

**DOI:** 10.3390/cancers14133129

**Published:** 2022-06-27

**Authors:** Anne Rupprecht, Ulrike Theisen, Franziska Wendt, Marcus Frank, Burkhard Hinz

**Affiliations:** 1 Institute of Pharmacology and Toxicology, Rostock University Medical Centre, 18057 Rostock, Germany; rupprechta31@gmail.com (A.R.); ulrike.theisen@med.uni-rostock.de (U.T.); franziska.wendt@med.uni-rostock.de (F.W.); 2 Electron Microscopy Centre, Rostock University Medical Centre, 18057 Rostock, Germany; marcus.frank@med.uni-rostock.de; 3 Department Life, Light and Matter, University of Rostock, 18059 Rostock, Germany

**Keywords:** cannabinoids, Δ^9^-tetrahydrocannabinol, cannabidiol, glioblastoma cells, mitochondria, oxygen consumption rate, electron transport chain complex proteins

## Abstract

**Simple Summary:**

Cannabidiol (CBD) is a phytocannabinoid from *Cannabis sativa* L. that exhibits no psychoactivity and, like the psychoactive cannabinoid Δ^9^-tetrahydrocannabinol (THC), shows anticancer effects in preclinical cell and animal models. Previous studies have indicated a stronger cancer-targeting effect when THC and CBD are combined. Here, we investigated how the combination of THC and CBD in a 1:1 ratio affects glioblastoma cell survival. The compounds were found to synergistically enhance cell death, which was attributed to mitochondrial damage and disruption of energy metabolism. A detailed look at the mitochondrial electron transfer chain showed that THC/CBD selectively decreased certain subunits of complexes I and IV. These data highlight the fundamental changes in cellular energy metabolism when cancer cells are exposed to a mixture of cannabinoids and underscore the potential of combining cannabinoids in cancer treatment.

**Abstract:**

Phytocannabinoids represent a promising approach in glioblastoma therapy. Previous work has shown that a combined treatment of glioblastoma cells with submaximal effective concentrations of psychoactive Δ^9^-tetrahydrocannabinol (THC) and non-psychoactive cannabidiol (CBD) greatly increases cell death. In the present work, the glioblastoma cell lines U251MG and U138MG were used to investigate whether the combination of THC and CBD in a 1:1 ratio is associated with a disruption of cellular energy metabolism, and whether this is caused by affecting mitochondrial respiration. Here, the combined administration of THC and CBD (2.5 µM each) led to an inhibition of oxygen consumption rate and energy metabolism. These effects were accompanied by morphological changes to the mitochondria, a release of mitochondrial cytochrome c into the cytosol and a marked reduction in subunits of electron transport chain complexes I (NDUFA9, NDUFB8) and IV (COX2, COX4). Experiments with receptor antagonists and inhibitors showed that the degradation of NDUFA9 occurred independently of the activation of the cannabinoid receptors CB_1_, CB_2_ and TRPV1 and of usual degradation processes mediated via autophagy or the proteasomal system. In summary, the results describe a previously unknown mitochondria-targeting mechanism behind the toxic effect of THC and CBD on glioblastoma cells that should be considered in future cancer therapy, especially in combination strategies with other chemotherapeutics.

## 1. Introduction

Over the past two decades, a large number of extensive preclinical studies have shown that cannabinoids of plant, synthetic and endogenous origin can inhibit tumourigenesis and tumour spread (for review [1,2]). Studies on the effect of cannabinoids on glioma cells have been published particularly frequently, and are of great importance in that glioblastoma multiforme or astrocytoma grade IV, an extremely aggressive cancer, has a high resistance to radiation and standard chemotherapy (for review [3]). In this context, the pivotal description of the tumour-regressive effect of the major and psychoactive phytocannabinoid Δ^9^-tetrahydrocannabinol (THC) and the synthetic cannabinoid WIN 55,212-2 on glioma xenografts from rats and mice represented the first comprehensive study of this century to investigate cannabinoids as potential anticancer drugs [4]. Soon after, a growth inhibitory effect on glioma xenografts was confirmed for the selective cannabinoid receptor CB_2_ agonist JWH-133 [5]. In the meantime, cannabinoid-mediated anticancer effects have also been demonstrated in a variety of other tumour entities, whereby the effects shown here range from the initially demonstrated inhibition of proliferation and induction of tumour cell apoptosis to proautophagic, anti-angiogenic, anti-invasive and antimetastatic effects (for review see [1,2]).

An interesting new aspect of cannabinoid cancer research was initiated with the finding that THC and the non-psychoactive phytocannabinoid cannabidiol (CBD) synergistically inhibit glioblastoma cell proliferation [6], raising the possibility of improved overall efficacy for future clinical trials. Soon after, local administration of submaximal effective doses of THC and CBD at a 1:1 ratio was shown to synergistically increase the tumour-regressive effect of temozolomide, a standard chemotherapeutic agent for glioblastoma therapy, on glioma xenografts from nude mice [7]. Later, the same group found strong anticancer effects in subcutaneous and intracranial glioma cell-derived tumour xenografts after oral administration of a 1:1 THC/CBD combination and temozolomide [8,9]. Other work demonstrated an increased radiosensitivity in glioma cells and in an orthotopic mouse glioma model with regard to the viability of tumour cells or the reduction of tumour volume when pretreatment with a combination of THC and CBD was performed [10]. Finally, this research culminated in a phase Ib randomised placebo-controlled trial published in 2021 in which nabiximols oromucosal cannabinoid spray (standardised extract of *Cannabis sativa* L. with an approximate 1:1 ratio of THC and CBD) was administered to temozolomide-treated patients with recurrent glioblastoma [11]. This study impressively showed a 1-year survival rate of 83% in the nabiximols group versus a survival rate of 44% in the placebo group, with the authors suggesting further investigation in an adequately powered randomised controlled trial.

Except for general parameters such as tumour cell apoptosis [6,7], viability [6,7,10] and tumour volume [7], the molecular mechanisms of the antiglioma effect caused by the cannabinoid combination have not yet been sufficiently investigated. A worthwhile research area in this regard is cellular energy metabolism, which has recently been presented as a promising target for glioblastoma therapy [12]. As a matter of fact, CBD mediates a variety of actions on mitochondrial functions (for review see [13]). Divergent effects on mitochondrial basal respiration with increases in mammary carcinoma cells [14] and varying degrees of inhibition in prostate carcinoma [14], gastric cancer [15], colorectal cancer [16], neuroblastoma [17], glioma [18] and hepatocellular carcinoma cells [19] were registered. However, a uniform pattern cannot be given here, especially as the concentrations tested varied greatly and very high CBD concentrations (i.e., ≥5 µM) were mostly used in these studies [14,17,18,19]. In addition, the importance of corresponding further investigations is underlined by the fact that so far there are only sporadic reports on the effect of cannabinoids on the individual proteins of the complexes of the mitochondrial electron transport chain (ETC).

Here, using the glioblastoma cell lines U251MG and U138MG, we show for the first time that the combination of THC and CBD in a 1:1 ratio leads to a significant decrease in the oxygen consumption rate (OCR), an established parameter of mitochondrial function, associated with a decrease in proteins of ETC complexes, mainly I and IV. These effects were registered with a submaximal toxic THC/CBD combination (2.5 µM each) and thus offer good starting points for further investigation of combination strategies with established or currently preclinically tested chemotherapeutic agents.

## 2. Materials and Methods

### 2.1. Materials

(-)-trans-Δ^9^-THC (THC, #THC-135-10LE) was purchased from Lipomed AG (Arlesheim, Switzerland). Cannabidiol (CBD, #BN0124) was bought from Biotrend Chemikalien GmbH (Cologne, Germany). Bafilomycin A_1_ (#Cay11038-500) and leupeptin were obtained from Biomol GmbH (Hamburg, Germany). Tin protoporphyrin IX dichloride (SnPPIX, #ALX-430-051-M005) was purchased from Enzo Life Sciences (Lörrach, Germany). Bortezomib (#sc-217785) was bought from Santa Cruz Biotechnology, Inc. (Heidelberg, Germany). Aprotinin, bromophenol blue, hydrogen peroxide solution (H_2_O_2_, 30%), luminol, MG-132 (#474790), orthovanadate, p-coumaric acid and phenylmethanesulfonyl fluoride (PMSF) were obtained from Sigma-Aldrich Corporation (Taufkirchen, Germany). Aqua ad iniectabilia was bought from Braun Melsungen AG (Melsungen, Germany). Methanol was purchased from J. T. Baker (Griesheim, Germany). Acetic acid, dimethyl sulfoxide (DMSO), ethylenediaminetetraacetic acid (EDTA), glycerin, glycine, hydrochloric acid 37% (HCl), Ponceau S, sodium chloride (NaCl), sodium hydroxide (NaOH), sodium dodecyl sulfate (SDS) ultra-pure, Tris ultrapure and Tris hydrochloride (Tris HCl) were purchased from AppliChem (Darmstadt, Germany). 4-(2-Hydroxyethyl)-1-piperazineethanesulfonic acid (HEPES) and β-mercaptoethanol were obtained from Ferak Berlin GmbH (Berlin, Germany). Acrylamide (Rotiphorese^®^ Gel, 30%), albumin (IgG-free), ammonium peroxydisulphate (APS), Crystal Violet, N,N,N′,N′-tetramethylethylenediamine (TEMED), Triton^®^ X-100 and Tween^®^ 20 were obtained from Carl Roth GmbH + Co. KG (Karlsruhe, Germany). Non-fat milk (NFM) powder was bought from Bio-Rad Laboratories GmbH (Munich, Germany). Gibco^TM^ penicillin-streptomycin and Gibco^TM^ trypsin-EDTA were bought from Thermo Fisher Scientific Inc. (Schwerte, Germany). Dulbecco’s phosphate-buffered saline (DPBS) and fetal bovine serum (FBS) were obtained from PAN-Biotech GmbH (Aidenach, Germany).

### 2.2. Cell Culture

The human glioblastoma cell line U251MG (#09063001; RRID:CVCL_0021) was purchased from Sigma-Aldrich Corporation. The human glioblastoma cell line U138MG cell line (#ACC 291; RRID: CVCL_0020) was bought from DSMZ (Deutsche Sammlung von Mikroorganismen und Zellkulturen GmbH, Braunschweig, Germany). Both cell lines were cultured in Dulbecco’s modified eagle medium (DMEM) with 4.5 g/L glucose and UltraGlutamine I (Lonza Cologne GmbH, Cologne, Germany). DMEM was supplemented with 10% heat-inactivated FBS, 100 U/mL penicillin and 100 μg/mL streptomycin. The cells were cultured in a humidified incubator at 37 °C and 5% CO_2_.

All cell treatments with specific substances were performed in serum-free DMEM supplemented with 100 U/mL penicillin and 100 μg/mL streptomycin, applied after washing the cells with DPBS. The test substances were dissolved in absolute ethanol (THC, CBD), DMSO (AM-251, AM-630, bafilomycin A_1_, bortezomib, capsazepine and MG-132) or 1 M NaOH (SnPPIX). The final concentration of solvents in the incubation media of cells treated with the test substance and vehicle varied between experiments, but in no case exceeded 0.033% (*v*/*v*) for ethanol, 0.1% (*v*/*v*) for DMSO and 0.4 mM for NaOH. All incubation media of an experiment also contained the same amount of solvent.

### 2.3. Cellular Viability Assay

For viability analyses, two different assays were employed. In all experiments, U251MG and U138MG cells were seeded in 96-well plates at a density of 5000 cells per well in serum-containing DMEM and cultured for 24 h before washing with DPBS and treatment with the test substances in serum-free and phenol red free Gibco^TM^ DMEM with 4.5 g/L glucose and L-glutamine (#21063; Thermo Fisher Scientific Inc.) supplemented with 100 U/mL penicillin and 100 μg/mL streptomycin for 24 h. The use of serum-free media minimises loss of substance due to interaction with serum proteins and discrepancies in cell number over time due to different proliferation rates of cell lines.

The metabolic activity of the cells was determined using WST-1 reagent (Roche Diagnostics, Mannheim, Germany). The water-soluble tetrazolium WST-1 was added to a final dilution of 1:10 and cells were further incubated for 10 min before the absorbance was measured at 450 nm (wavelength correction at 690 nm) using a microplate reader.

For cell number analysis, cells were fixed with ice-cold absolute ethanol for 30 min before incubation with crystal violet staining solution (0.1% [*w*/*v*] crystal violet in 10% ethanol) for 30 min. After thoroughly washing off the excess dye, the stained cells were dissolved with 10% acetic acid and the dye intensity was measured at 570 nm with a microplate reader.

### 2.4. Cellular Apoptosis Assay

The analysis of caspase-3 and -7 activity was carried out using Caspase-Glo^®^ 3/7 Assay (#G8093; Promega, Walldorf, Germany). For this purpose, U251MG and U138MG cells were seeded in 96-well plates at a density of 5000 cells per well in serum-containing DMEM for 24 h. Cells were then washed with DPBS and incubated with the appropriate test substances for a further 24 h in serum-free and phenol red free DMEM. Caspase-Glo^®^ 3/7 Reagent was added in equal volume proportions according to the manufacturer’s instructions and incubated in the dark for 1 h. Luminescence was measured with a microplate reader.

### 2.5. Seahorse XFe Analysis

The oxygen consumption rate (OCR) and extracellular acidification rate (ECAR) were determined using a Seahorse XFe24 Analyser (Agilent Technologies, Inc., Waldbronn, Germany) according to the manufacturer’s instructions. U251MG and U138MG cells were seeded on Seahorse 24XFe plates (Agilent Technologies, Inc., Waldbronn, Germany) at a density of 25,000 cells per well and incubated in serum-containing DMEM for 24 h. After washing with DPBS, the cells were treated with the test substances in serum-free DMEM for 24 h. To start the Seahorse XFe analysis, media were changed on the cells to unbuffered XF base media, pH 7.4 (#103575-100, Agilent Technologies, Inc.) supplemented with 10 mM glucose, 2 mM glutamine and 1 mM pyruvate and incubated for 1 h. Compounds from the Seahorse XF Cell Mito Stress Test Kit (#133015-100, Agilent Technologies, Inc.) were added to the wells at final concentrations according to the manufacturer’s instructions (port A: 1.5 μM oligomycin (ATP synthase inhibitor), port B: 1.0 μM FCCP (carbonyl cyanide-4 (trifluoromethoxy) phenylhydrazone; uncoupling agent that collapses the proton gradient and disrupts the mitochondrial membrane potential), port C: 0.5 μM rotenone (complex I inhibitor) and 0.5 μM antimycin A (complex III inhibitor)). OCR and ECAR per well were normalised to the amount of protein determined after the experiment. To this end, 10 µL of lysis buffer (50 mM HEPES, 150 mM NaCl, 1 mM EDTA, 1% [*v*/*v*] Triton^®^ X-100, 10% [*v*/*v*] glycerol, 10 µg/mL aprotinin, 1 µg/mL leupeptin, 1 mM orthovanadate and 1 mM PMSF) was added to each well and the lysates were collected. Protein concentration was determined using the Pierce™ BCA (bicinchoninic acid) Protein Assay Kit (Thermo Fisher Scientific Inc.). Calculation of basal respiration, ATP production-based respiration, spare capacity and proton leak was performed using the Seahorse XF Cell Mito Stress Test Report Generator (Agilent Technologies, Inc.).

### 2.6. Lactate Release Analysis

Lactate was determined in the supernatant of the cultured cells using the L-Lactate Assay Kit (#ab65330, Abcam, Cambridge, UK) according to the manufacturer’s instructions. Cell culture supernatants were obtained from U251MG cells seeded at a density of 25,000 cells per well in 48-well plates in 10% FBS containing DMEM for 24 h, which were washed with DPBS and subsequently treated with the test substances in serum-free DMEM for 24 h. Lactate concentrations were measured at 570 nm (reference wavelength 450 nm) in a microplate reader.

### 2.7. Total Cellular Protein Isolation

U251MG or U138MG cells were seeded in 6-well plates at a density of 200,000 cells per well and cultured in DMEM containing 10% heat-inactivated FBS for 24 h before treatment with substances in serum-free DMEM. After the respective incubation time, the medium supernatant was separated from the cells, and the cells were washed with DPBS, detached by trypsin-EDTA and collected again in the medium supernatant. Cells were harvested in a pellet after centrifugation for 5 min (200× *g*, 4 °C). The cell pellet was washed with DPBS and collected again after centrifugation (250× *g*, 4 °C) for 5 min. Lysis buffer (50 mM HEPES, 150 mM NaCl, 1 mM EDTA, 1% [*v*/*v*] Triton^®^ X-100, 10% [*v*/*v*] glycerol, 10 µg/mL aprotinin, 1 µg/mL leupeptin, 1 mM orthovanadate and 1 mM PMSF) was added to the cell pellet, followed by 30 min incubation on ice and subsequent centrifugation for 5 min (20,817× *g*, 4 °C). The resulting supernatant, containing total cellular protein, was collected and stored for further protein analysis. Protein concentration was measured using the Pierce™ BCA Protein Assay Kit (Thermo Fisher Scientific Inc.).

### 2.8. Mitochondrial Protein Isolation

U251MG or U138MG cells were seeded, treated and harvested as described in the whole cellular protein isolation experiments. The resulting cell pellet was washed in 0.9% NaCl and centrifuged for 5 min (500× *g*, 4 °C). To isolate mitochondria and to obtain the cytosolic protein fraction, the Qproteome Mitochondria Isolation Kit (Qiagen GmbH, Hilden, Germany) was used according to the manufacturer’s instructions for standard preparation. For extraction of mitochondrial protein, the resulting mitochondrial fraction was treated with lysis buffer, centrifuged and collected in a similar manner to the protocol listed for isolation of total cellular protein.

### 2.9. Western Blot Analysis

Equal amounts of protein were separated on a 15% SDS-polyacrylamide gel, transferred to a nitrocellulose membrane and incubated for 1 h in 5% (*w*/*v*) NFM in Tris-buffered saline containing 0.1% (*v*/*v*) Tween^®^ 20 (TBS-T buffer). After washing with TBS-T buffer, membranes were incubated overnight at 4 °C with primary antibodies in 1% (*w*/*v*) NFM. The following antibodies were purchased from Thermo Fisher Scientific Inc.: NDUFA9 (#459100, RRID:AB_2532223), OxPhos Complex IV subunit IV (#A21348; COX4, RRID:AB_2535839), ATP Synthase β subunit (#A21351; ATP5B, RRID:AB_221512) and OxPhos Human WB Antibody Cocktail (#45-8199, RRID:AB_2533836). Other antibodies were LC3A/B (#4108, RRID: AB_2137703) and Cytochrome c (#11940, RRID:AB_2637071), both from Cell Signaling Technology (Frankfurt/Main, Germany), SDHA (#ab14715, RRID:AB_301433) and VDAC (#ab14734, RRID:AB_443084) from Abcam (Berlin, Germany), HO-1 (#ADI-SPA-894F, RRID:AB_10631417) from Enzo Life Sciences and GAPDH (#G9545, RRID:AB_796208), β-actin (#A5441, RRID:AB_476744) and LONP1 (#HPA002192, RRID:AB_1079695) from Sigma-Aldrich Corporation. After washing with TBS-T buffer, membranes were incubated with secondary horseradish peroxidase-coupled antibodies (anti-rabbit antibody, #7074, RRID:AB_2099233; anti-mouse antibody, #7076, RRID:AB_330924; Cell Signaling Technology) in 1% (*w*/*v*) NFM in TBS-T buffer for 1 h at room temperature. To visualise antibody binding, a substrate solute for chemiluminescence detection (100 mM Tris hydrochloride, pH 8.5; 1.25 mM luminol; 200 µM p-coumaric acid; 0.09% [*v*/*v*] H_2_O_2_) was added and signal detection was performed using the ChemiDoc XRS gel documentation system manufactured by Bio-Rad Laboratories GmbH (Munich, Germany). Quantification of signal intensity was carried out using the 1-D analysis software Quantity One (Bio-Rad Laboratories GmbH). The signal of a specific protein band was normalised to the signal of the loading control. Accordingly, the protein levels of VDAC for mitochondrial proteins, GAPDH for cytosolic proteins or β-actin for total cellular protein membranes were also analysed. Protein levels were then calculated relative to the vehicle control. The Precision Plus Protein^TM^ Dual Colour Standard from Bio-Rad Laboratories GmbH was used to identify the molecular weight of the bands. The Western blots shown in the Figures are included in File S1 in uncropped form.

### 2.10. Electron Microscopy

For electron microscopy, U251MG or U138MG cells were seeded at a density of 200,000 cells per well in a 6-well plate and cultured in DMEM containing 10% heat-inactivated FBS for 24 h before treatment with substances in serum-free DMEM. Cells were then incubated with THC/CBD or vehicle for 24 h and subsequently treated in the same way as described for the protein extraction experiments. The cell pellets were fixed in a solution containing 2% glutaraldehyde and 1% paraformaldehyde in 0.1 M phosphate buffer pH 7.3, and were stored at 4 °C until further processing for embedding. Next, after two washes in 0.1 M phosphate buffer, the cells were mixed with prewarmed 2% low melting agarose (Sigma-Aldrich Corporation) in 0.05 M HEPES buffer at 40 °C, collected by centrifugation and enclosed as a pellet in the agarose after hardening. The specimen blocks were then post-fixed in a solution of 1% osmium tetroxide (Carl Roth GmbH + Co. KG, Karlsruhe, Germany) for 2 h and, after washes in distilled water, were dehydrated in an ascending series of acetone. Resin infiltration was started with an overnight incubation in a 1:1 mixture of acetone and Epon resin (48% Epon 812, 30% methylnadic anhydride, 20.7% 2-dodecenylsuccinic acid anhydride and 1.3% 2,4,6-Tris(dimethylaminomethyl)phenol; all components from Serva, Heidelberg, Germany) followed by pure Epon resin for 4 h. Specimen blocks were transferred to silicone rubber moulds with fresh resin and were cured in an oven at 60 °C for 2 days. Further processing included trimming of the resin blocks (Leica EM Trim2, Leica Microsystems, Wetzlar, Germany) and subsequent sectioning on an ultramicrotome (Ultracut S, Reichert, Wien, Austria) using a diamond knife (Diatome, Nidau, Switzerland). Semithin sections with a thickness of 0.5 µm were stained with toluidine blue to visualise and select the areas for ultrastructural inspection. Ultrathin sections of approximately 50–70 nm thickness were transferred to copper grids and stained with uranyl acetate and lead citrate prior to examination with a Zeiss EM902 transmission electron microscope operated at 80 kV (Carl Zeiss, Oberkochen, Germany). Digital images were acquired with a side-mounted 1x2k FT-CCD Camera (Proscan, Scheuring, Germany) using iTEM camera control and imaging software (Olympus Soft Imaging Solutions, Münster, Germany).

For quantification of mitochondrial density, all mitochondria were manually outlined in the images of the greatest magnification and the mean intensity measured with the open source software FIJI (https://imagej.net/software/fiji). In order to present the data as density, the measured intensities were subtracted from the maximal value of 65,535 which represents white in 16-bit images. The data were plotted as mean over all data points ± SEM.

### 2.11. Quantitative Reverse Transcriptase Polymerase Chain Reaction (RT-PCR)

U251MG or U138MG cells were seeded at 200,000 cells per well in a 6-well plate, cultured for 24 h in DMEM containing 10% heat-inactivated FBS, treated with compounds for another 24 h in serum-free DMEM, and then harvested as in the protein isolation experiments. Total RNA was isolated from the obtained cell pellet using the RNeasy Mini Kit from Qiagen GmbH (Hilden, Germany). For quantification of TFAM, the Applied Biosystems^®^ TaqMan^®^ Gene Expression Assay (Assay ID: Hs00273372_s1) and the Applied Biosystems^®^ TaqMan^®^ RNA-to-CT™ 1-Step Kit from Thermo Fisher Scientific Inc. were used according to the manufacturer’s instructions. Peptidylprolyl isomerase A (PPIA; Assay ID: Hs99999904_m1) was used as the housekeeping gene to normalise TFAM mRNA levels prior to comparison with the respective vehicle controls.

### 2.12. Statistics

All statistical analyses were performed using GraphPad Prism 9.3.0 (GraphPad Software, Inc., San Diego, CA, USA). Student’s unpaired two-tailed *t* tests were carried out to compare two groups. Comparisons between more than two groups were conducted by one-way ANOVA with Dunnett’s post hoc test when all conditions were compared with a vehicle control or by Bonferroni’s post hoc test for selected group comparisons.

## 3. Results

### 3.1. Combined Treatment with THC and CBD Leads to a Viability-Reducing and Proapoptotic Effect on Human Glioblastoma Cells

The studies on the effects of THC and CBD were conducted using the established human glioblastoma cell lines U251MG and U138MG, which differ in their resistance to temozolomide [20]. In contrast, both cell lines show similarities in terms of their metabolism and mitochondrial homeostasis by exhibiting similar expression levels of the master regulator of mitochondrial biogenesis, peroxisome proliferator-activated receptor-γ coactivator-1α (PGC-1α) [21]. The comparison of results obtained in these two cell lines therefore allows us to infer which effects are likely to occur in all glioblastomas, independent of tumour resistance to a common chemotherapeutic.

**Figure 1:**
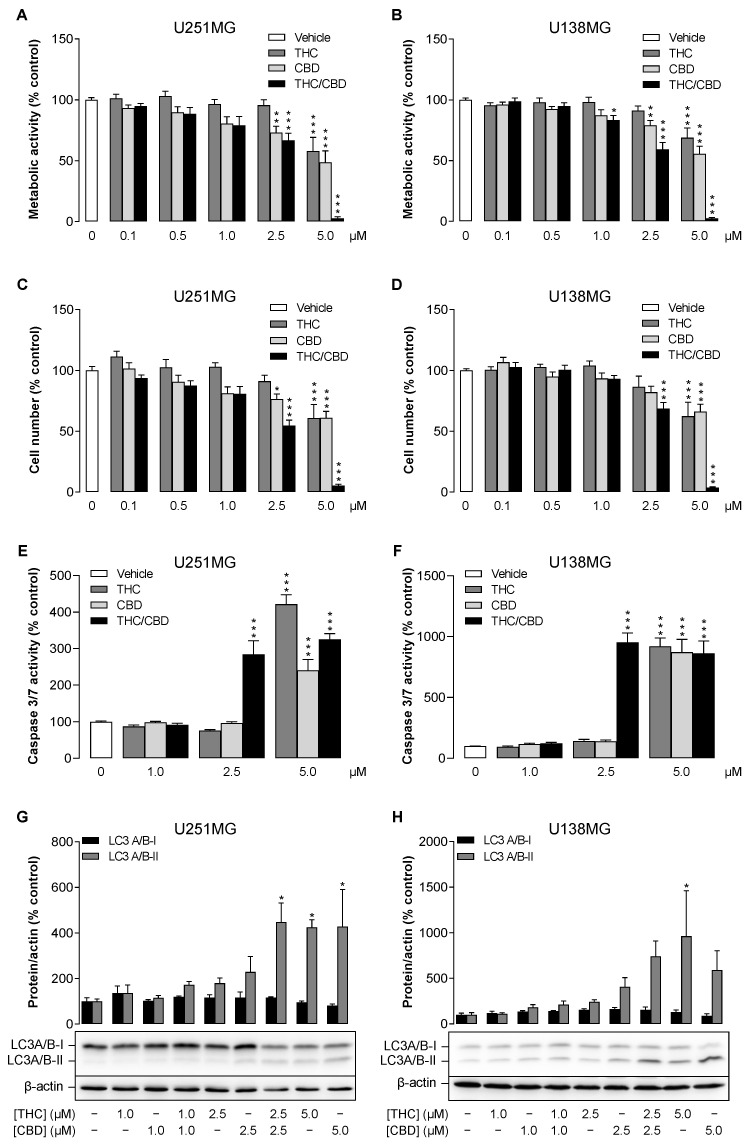
Influence of THC and CBD, administered alone or in combination, on metabolic activity, viability, apoptosis and autophagy in human glioblastoma cells. U251MG and U138MG cells were incubated for 24 h with selected concentrations of THC and CBD or their combination (in a ratio of 1:1) as well as vehicle control. Thereafter, metabolic activity was determined by WST-1 assay (**A**,**B**), cell number by crystal violet staining (**C**,**D**), caspase-3/-7 activity by ELISA (**E**,**F**) and LC3-I to LC3-II conversion by Western blot analysis (**G**,**H**). All percentage values shown refer to the vehicle control, which was set to 100%. The data represent mean values ± SEM of *n* = 10–12 (4 independent experiments in (**A**–**E**)), *n* = 9 (3 independent experiments in **F**) and *n* = 3 or *n* = 4 independent experiments in (**G**,**H**). * *p* ≤ 0.05, ** *p* ≤ 0.01, *** *p* ≤ 0.001 vs. corresponding vehicle control, one-way ANOVA with Dunnett’s post hoc test.

In a first approach, the impact of THC and CBD on the viability and metabolic activity of these cells was measured with the colorimetric WST-1 assay. As shown in Figure 1A,B, THC and CBD did not cause a strong reduction in metabolism in either cell line when administered individually at up to 1 µM each or in combination (up to 1 µM of each cannabinoid). A highly significant inhibitory effect (*p* ≤ 0.01) on viability was observed for 2.5 µM CBD and for 5.0 µM THC and CBD when applied individually. Thereby, a distinct synergistic effect could be registered with a combination of 5 µM THC and CBD each.

A comparable effect was found in the cell number analysis using crystal violet staining shown in Figure 1C,D. In agreement with the almost complete inhibition of metabolic activity, a nearly total cell loss was registered after incubation of either cell line with the combination of 5 µM THC and CBD.

Analysis of effector caspases-3 and -7 by specific ELISA confirmed the induction of apoptosis and supported the synergistic effect of the combination of 2.5 µM THC and CBD (Figure 1E,F).

To investigate autophagy as another possible synergistic effect of THC and CBD, the conjugation of microtubule associated protein 1 light chain (LC3-I) with phosphatidylethanolamine (PE) to LC3-II, which is responsible for the maturation of autophagosomes [22,23], was next addressed. Determination of LC3-I and LC3-II concentrations by Western blot showed significant (Figure 1G) or measurable (Figure 1H) upregulation of LC3-II in the investigated cell lines, whereby at least in U251MG cells a synergistic effect of the combination at 2.5 µM THC and CBD was evident compared to the effect of the individual substances. Activation of autophagy was assessed by the expression of phosphatidylethanolamine-conjugated LC3A/B-II protein normalised to β-actin instead of the protein ratio between LC3-I and LC3-II, as different affinities of antibodies against LC3-I and LC3-II and different expression levels of these proteins depending on cell line and tissue have been reported in the literature [23,24].

### 3.2. Combined Treatment with THC and CBD Suppresses Mitochondrial Respiration in Human Glioblastoma Cells, but Triggers Cell Line-Specific Differential Effects on Glycolysis

**Figure 2:**
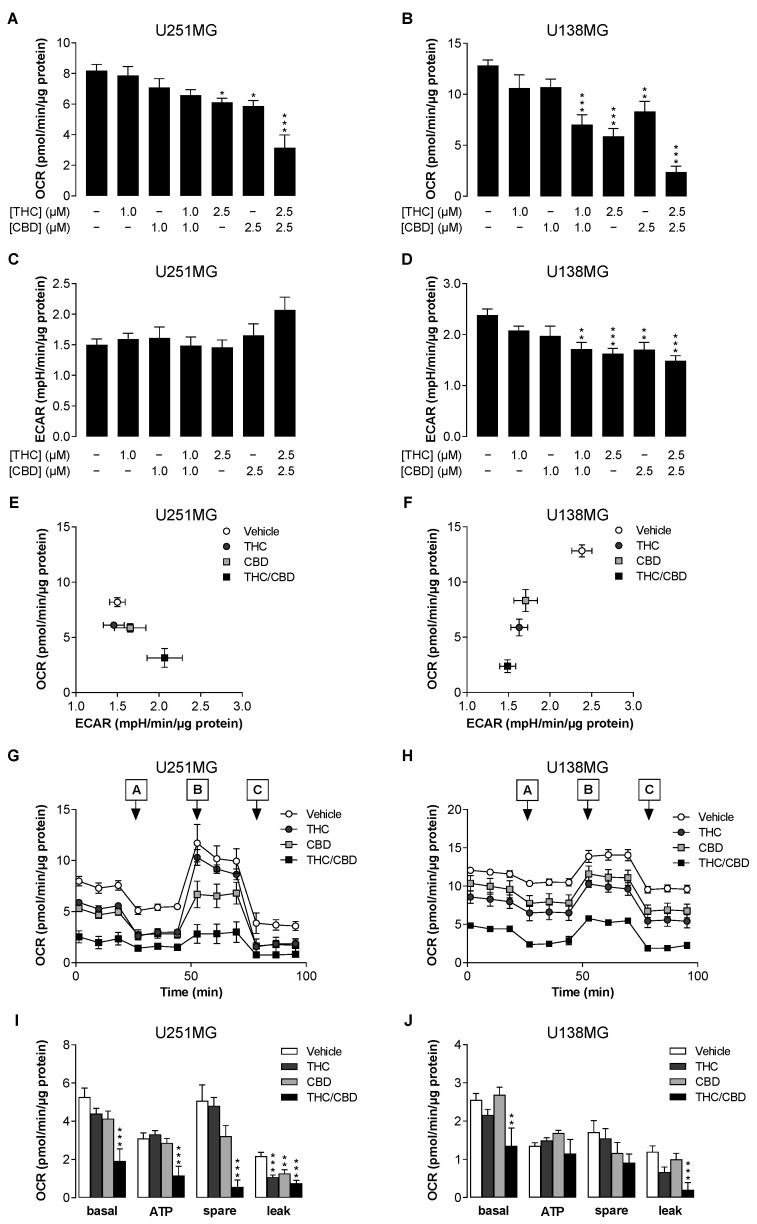
Influence of THC and CBD, administered alone or in combination, on oxygen consumption rate (OCR) and extracellular acidification rate (ECAR) of human glioblastoma cells. U251MG and U138MG cells were incubated with selected concentrations of THC and CBD, their combination (in a ratio of 1:1) or the vehicle control for 24 h. Thereafter, OCR (**A**,**B**) and ECAR (**C**,**D**) were determined using the Seahorse XFe24 Analyser. The effects of selected treatment groups on OCR and ECAR are summarised in the energy maps in panels (**E**,**F**). Subsequently, a mitochondrial stress test was performed. For this purpose, the sensor cartridges were loaded at the indicated times with 1.5 µM oligomycin (port A), 1.0 µM FCCP (port B) and 0.5 µM each of antimycin A and rotenone (port C). Panels (**G**,**H**) show representative time courses of OCR in both cell lines treated with 2.5 µM THC and/or CBD. Panels (**I**,**J**) present calculations of basal respiration (basal), ATP-bound respiration (ATP), reserve respiratory capacity (spare) and proton leak. The data represent mean values ± SEM of *n* = 6–9 of 3 independent experiments per group (**A**–**F**,**I**,**J**), or *n* = 2–3 of 1 experiment per group (**G**,**H**). * *p* ≤ 0.05, ** *p* ≤ 0.01, *** *p* ≤ 0.001 vs. corresponding vehicle control, one-way ANOVA with Dunnett’s post hoc test.

According to the WST assay, the combination of THC and CBD led to decreases in the viability of the treated cells at the level of metabolic activity. In order to assess changes to the cellular energy metabolism upon treatment with THC/CBD, we next determined the oxygen consumption rate (OCR) as an indicator of mitochondrial respiration and the extracellular acidification rate (ECAR) as an indicator of glycolysis. Measurements carried out with the Seahorse analyser revealed an inhibition of OCR by THC and CBD (2.5 µM each) in both cell lines, as well as a much more pronounced reduction when the corresponding combination was applied (Figure 2A,B). For the combination of 1 µM THC and CBD each, a significant OCR reduction could also be detected in U138MG cells (Figure 2B), whereby this cell line showed a comparatively higher basal respiration rate in vehicle-treated cells compared to U251MG cells. Interestingly, a divergent pattern was found for glycolysis. Here, an increase of ECAR was observed in U251MG cells incubated with the combination of 2.5 µM each of THC and CBD (Figure 2C), while a significant decrease in ECAR was registered by the administration of THC and CBD alone (2.5 µM each) as well as by the combinations of 1 and 2.5 µM of the two cannabinoids (Figure 2D). Accordingly, plotting OCR versus ECAR on an energy map showed bioenergetic profiles for the combination of THC and CBD (2.5 µM each) with a strong reduction of the oxidative phosphorylation compared to the single substances (Figure 2E,F) and a simultaneously higher (U251MG, Figure 2E) or lower (U138MG, Figure 2F) glycolytic activity. The increased ECAR in U251MG cells may reflect a compensatory attempt by the cells to regulate energy metabolism in response to reduced OCR, which, however, had no positive effects on cell survival (Figure 1). To confirm these results, lactate secretion from U251MG cells was also measured, demonstrating a significant increase upon treatment with a combination of 2.5 µM each of THC and CBD (Figure S1).

To gain a more detailed insight into the mitochondrial function, a mitochondrial stress test was performed, which in addition to basal respiration also includes the parameters ATP-linked respiration, maximal and reserve capacities, and non-mitochondrial respiration. This showed a statistically significant synergistic inhibition of the basal respiration by the combination of THC and CBD (2.5 µM each) in both U251MG (Figure 2G,I) and U138MG cells (Figure 2H,J). In U251MG cells, which exhibit a high proportion of ATP-linked respiration as well as strong FCCP-induced maximal respiration under vehicle treatment, both ATP-linked respiration and especially reserve capacity were strongly reduced by the combination of THC and CBD. In contrast, U138MG cells displayed a high non-mitochondrial respiration and, under vehicle treatment, only a small ATP-linked respiration as well as a slight induction of the respiration by FCCP. In these cells, THC/CBD also induced changes in the ATP-linked respiration and reserve capacity; however, these did not reach statistical significance. Overall, although both cell lines show differences in their energy metabolism, the combination of THC and CBD causes a similar OCR reduction in either cell line, which is mainly due to the loss of mitochondrial respiration.

### 3.3. Combined Treatment with THC and CBD Confers Structural Mitochondrial Changes and Promotes the Release of Cytochrome c

**Figure 3:**
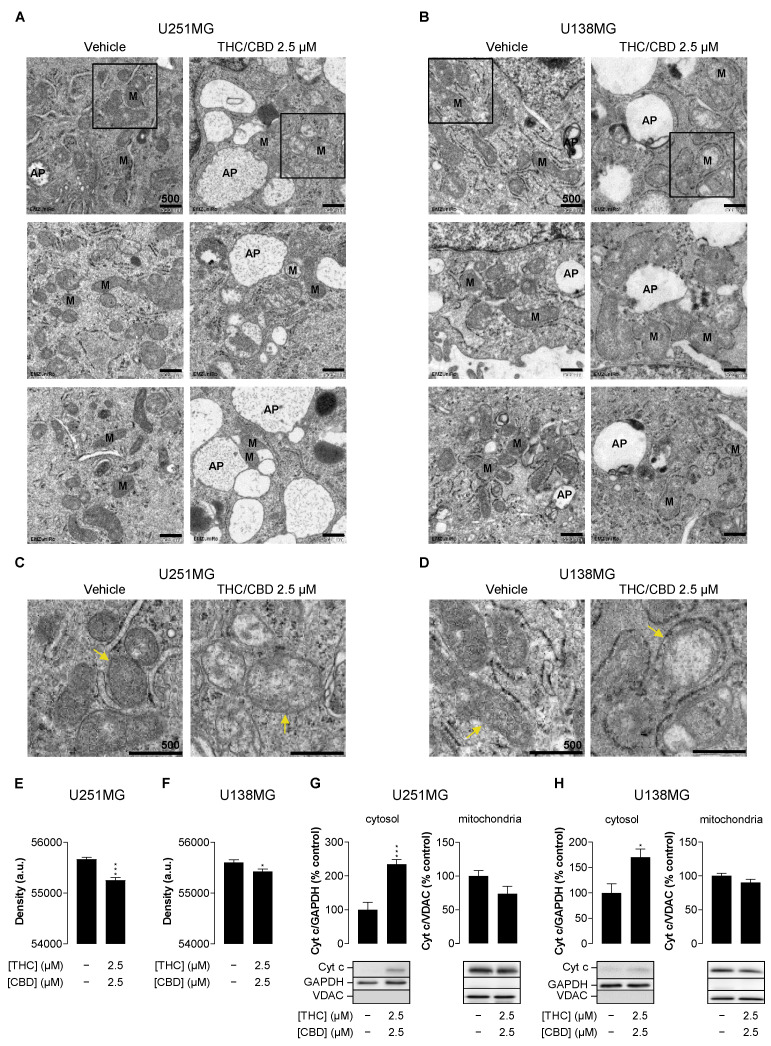
Influence of THC and CBD on mitochondrial structure and permeability of human glioblastoma cells. Representative images from transmission electron microscopy of U251MG (**A**) and U138MG cells (**B**) treated for 24 h with vehicle (left) or 2.5 µM THC/CBD (right). The marked structures correspond to mitochondria (M) and autophagosomes (AP), respectively. (**C**,**D**) are enlarged views of the outlined areas in (**A**,**B**), with yellow arrows indicating the mitochondria marked in the overview images. Mitochondrial density of all mitochondria per image was quantified as intensity value subtracted from maximal display value (**E**,**F**). Data were obtained from 7 images/108 mitochondria (vehicle), 7 images/58 mitochondria (THC/CBD, panel **E**), and 11 images/115 mitochondria (vehicle) or 8 images/74 mitochondria (THC/CBD, panel **F**), respectively. Cytochrome c (Cyt c) was determined by Western blot analysis in the cytosolic and isolated mitochondrial fraction (**G**,**H**). A representative Western blot and the quantitative analysis of *n* = 3–5 independent experiments are shown. The values correspond to the mean values ± SEM. * *p* ≤ 0.05, *** *p* ≤ 0.001 vs. corresponding vehicle control, unpaired two-tailed *t* test.

Reduced basal and ATP-linked respiration as well as spare and maximal capacities in THC/CBD-treated cells may be due to mitochondrial damage. To investigate this, electron microscopic examinations were carried out. The cytoplasm of cannabinoid-treated cells exhibited high vacuolisation (Figure 3A,B images on the right). Many vesicles were recognisable as phagosomes (Figure 3C,D), corresponding to the increase of autophagy upon treatment with THC/CBD shown before (Figure 1G,H). The proximity of phagosomes to mitochondria was prominent. The mitochondria in the THC/CBD-treated cells displayed marked enlargement as well as rounding, and the mitochondrial cristae showed marked structural damage (Figure 3C,D). Quantification revealed a significant loss of electron-dense material within the mitochondria of THC/CBD-treated cells as compared to the vehicle control (Figure 3E,F). Consistent with the microscopically detected mitochondrial damages, there was also a release of cytochrome c into the cytosol (Figure 3G,H). This release indicates a loss of mitochondrial membrane integrity and represents a known apoptosis triggering mechanism, consistent with the activation of effector caspases shown earlier (Figure 1E,F).

### 3.4. Combined Treatment with THC and CBD Mediates Downregulation of Specific ETC Proteins

**Figure 4:**
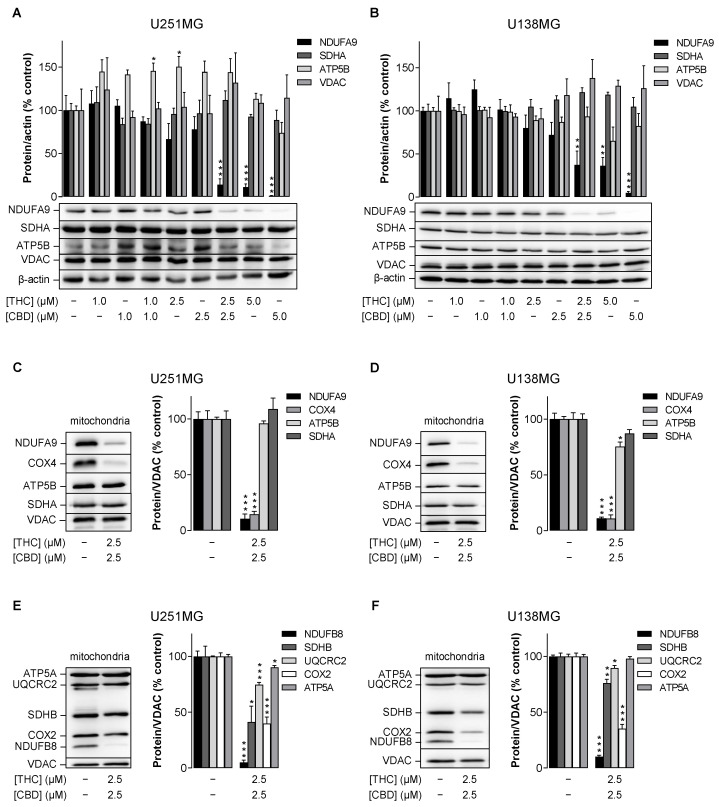
Influence of THC and CBD, administered alone or in combination, on protein levels of subunits of ETC complexes I (NDUFA9, NDUFB8), II (SDHA, SDHB), III (UQCRC2), IV (COX2, COX4) and V (ATP5A) in human glioblastoma cells. U251MG and U138MG cells were incubated with selected concentrations of THC and CBD or their combination (in a ratio of 1:1) for 24 h. Subsequently, cellular total protein extracts (**A**,**B**) or isolated mitochondrial fractions (**C**–**F**) were analysed for specific subunits of different ETC complexes by Western blots. All percentage values shown refer to the vehicle control, which was set to 100%. The values shown in the bar graphs are based on densitometric analyses of blots, whereby the specific subunits were normalised to β-actin (**A**,**B**) or VDAC (**C**–**F**). The blots shown are representative. The data in all panels are mean values ± SEM of *n* = 3–4 independent experiments per group. * *p* ≤ 0.05, ** *p* ≤ 0.01, *** *p* ≤ 0.001 vs. corresponding vehicle control, one-way ANOVA with Dunnett’s post hoc test (**A**,**B**) or unpaired two-tailed *t* test (**C**–**F**).

Mitochondrial damage is often associated with a defective ETC. To identify the cause of mitochondrial respiratory impairment and mitochondrial integrity, protein concentrations of mitochondrial proteins, especially ETC complexes, were examined. Accordingly, Western blot analyses were performed after treatment of cells with different concentrations of THC and CBD as well as the combination of both cannabinoids (Figure 4A,B). A highly significant downregulation of NADH:ubiquinone oxidoreductase subunit A9 (NDUFA9, subunit of ETC complex I) was obvious in both cell lines in the combination group (2.5 µM THC and CBD each) as well as after single treatment with 5 µM THC and CBD each (Figure 4A,B). Remarkably, under the same conditions, the protein concentrations of ATP synthase subunit β (ATP5B, subunit of complex V), succinate dehydrogenase complex, subunit A (SDHA, subunit of complex II) and voltage-dependent anion-selective channel (VDAC, channel of the outer mitochondrial membrane) remained unaffected (Figure 4A,B). Therefore, a general change in mitochondrial mass can be ruled out and a specific effect on NDUFA9 can be concluded.

To support these findings, Western blot analysis was performed on mitochondria isolated from cells after treatment with a combination of 2.5 µM THC and CBD (Figure 4C–F). Again, NDUFA9, but not ATP5B, SDHA and VDAC showed a strong decrease in protein levels (Figure 4C,D). On the other hand, a decrease in protein of about 90% was also observed for cytochrome c oxidase subunit 4 (COX4, subunit of complex IV) (Figure 4C,D), which was of a similar magnitude to the decrease observed for NDUFA9.

Corresponding regulation of subunits of complexes I and IV was also confirmed using an OxPhos antibody set. Thus, at the mitochondrial level, a significant reduction of the NADH:ubiquinone oxidoreductase subunit B8 (NDUFB8, subunit of complex I) as well as of the cytochrome c oxidase subunit 2 (COX2, subunit of complex IV) could be registered in the presence of the combination of THC and CBD (Figure 4E,F); this indicates a disruption of the respective entire protein complex. In addition, a moderate inhibition of succinate dehydrogenase complex subunit B (SDHB, subunit of complex II) and ubiquinol-cytochrome c reductase core protein 2 (UQCRC2, subunit of complex III) was observed, whereby the SDHB decrease in U251MG cells was even comparable to the decrease of COX2. On the other hand, ATP synthase subunit α (ATP5A, subunit of complex V) exhibited very little reduction under THC/CBD treatment of U251MG cells, which was not noted in U138MG cells (Figure 4E,F).

### 3.5. Downregulation of NDUFA9 by Combined Treatment with THC and CBD Is Independent of Cannabinoid Receptor Activation, Autophagy and Proteasomal Degradation

Since the greatest decrease in treatment with THC and CBD was in complex I, represented by NDUFA9, different pathways that might be involved in this effect were investigated next.

**Figure 5:**
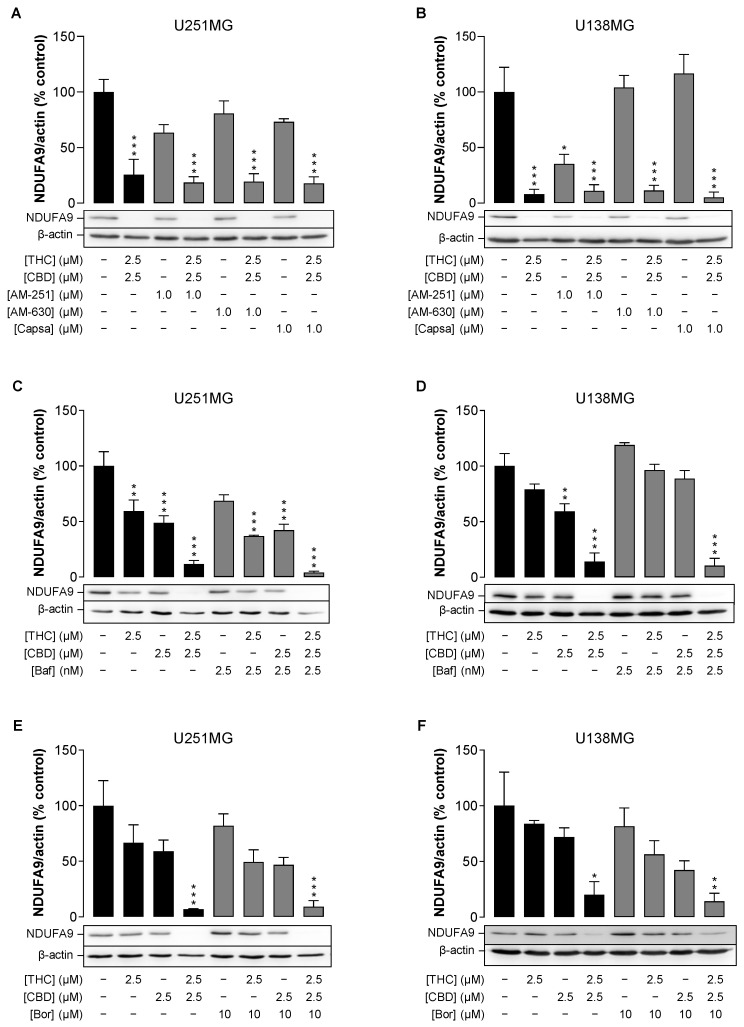
Role of cannabinoid-sensitive receptors (**A**,**B**), autophagy (**C**,**D**) and the proteasome (**E**,**F**) in downregulation of the mitochondrial subunit NDUFA9 by the combination of THC and CBD in human glioblastoma cells. U251MG and U138MG cells were incubated with selected concentrations of THC and CBD, their combination (in a ratio of 1:1) or vehicle control for 24 h. The receptor antagonists AM-251, AM-630 and capsazepine (1 µM each; panels (**A**,**B**)) were applied to the cells 1 h before the cannabinoids and inhibitors of autophagy (bafilomycin A_1_, 2.5 nM; panels **C**,**D**) or proteasome (bortezomib, 10 nM; panels (**E**,**F**)) were applied together with the cannabinoids. Antagonists and inhibitors were present throughout the incubation with the cannabinoids. Subsequently, cellular total protein extracts were analysed for NDUFA9 levels by Western blots. All percentage values shown refer to the vehicle control, which was set to 100%. The values shown in the bar charts are based on densitometric analyses of blots, whereby the NDUFA9 levels were normalised to β-actin. The blots shown are representative. The data in all panels are mean values ± SEM of *n* = 3 independent experiments per group. * *p* ≤ 0.05, ** *p* ≤ 0.01, *** *p* ≤ 0.001 vs. corresponding vehicle control, one-way ANOVA with Bonferroni’s post hoc test.

First, the identification of the upstream trigger for the loss of complex I was addressed. To this end, a possible involvement of the cannabinoid receptors CB_1_ and CB_2_ and transient receptor potential vanilloid 1 (TRPV1) in the effect of the combination of THC and CBD was investigated using antagonists against CB_1_ (AM-251), CB_2_ (AM-630) and TRPV1 (capsazepine). The receptor antagonists were used at concentrations of 1 µM, which were previously shown to inhibit the activation of CB_1_, CB_2_ and TRPV1 [25,26,27,28]. With the exception of AM-251, which on its own led to a reduction in NDUFA9 that was significant in U138MG cells, AM-630 and capsazepine exhibited no marked effect on basal NDUFA9 expression (Figure 5A,B). More importantly, none of the antagonists showed an inhibitory effect on the downregulation of NDUFA9 induced by the combination of THC and CBD, so that a receptor-independent effect of the combination can be assumed. 

Next, the role of autophagy in the reduction of complex I after THC/CBD exposure was investigated, as autophagy often correlates with mitochondrial damage [29,30]. However, simultaneous treatment of cells with THC/CBD and the late-phase autophagy inhibitor bafilomycin A_1_ had no effect on NDUFA9 levels (Figure 5C,D), suggesting that autophagy occurs downstream of complex I loss.

Finally, a possible role of the ubiquitin-proteasome system was addressed, which may exert both protective and detrimental effects on mitochondrial biogenesis, including complex I [31]. Corresponding inhibitor experiments were carried out with the proteasome inhibitor bortezomib, an N-protected dipeptide whose boron atom binds with high affinity and specificity to the 26S proteasome [32]. As shown in Figure 5E,F, however, the synergistic inhibitory effect of the combination of THC and CBD could neither be abolished nor partially blocked in this way. In the same context, MG-132, another potent and cell-permeable proteasome inhibitor, also showed no effect on cannabinoid-induced NDUFA9 decline in U251MG cells (Figure S2). Since MG-132 also inhibits LONP1 [33], a mitochondrial protease that has been reported to be activated in response to stress [34], this result further suggests that LONP1 is not involved in NDUFA9 turnover. This view is also supported by the absence of cannabinoid-induced upregulation of LONP1 (Figure S3A,B).

Overall, it seems unlikely that induced protein degradation is the cause of the reduced NDUFA9 levels after treatment with THC/CBD.

### 3.6. Combined Treatment with THC and CBD Upregulates the Expression of Heme Oxygenase-1 (HO-1) in Human Glioblastoma Cells

**Figure 6:**
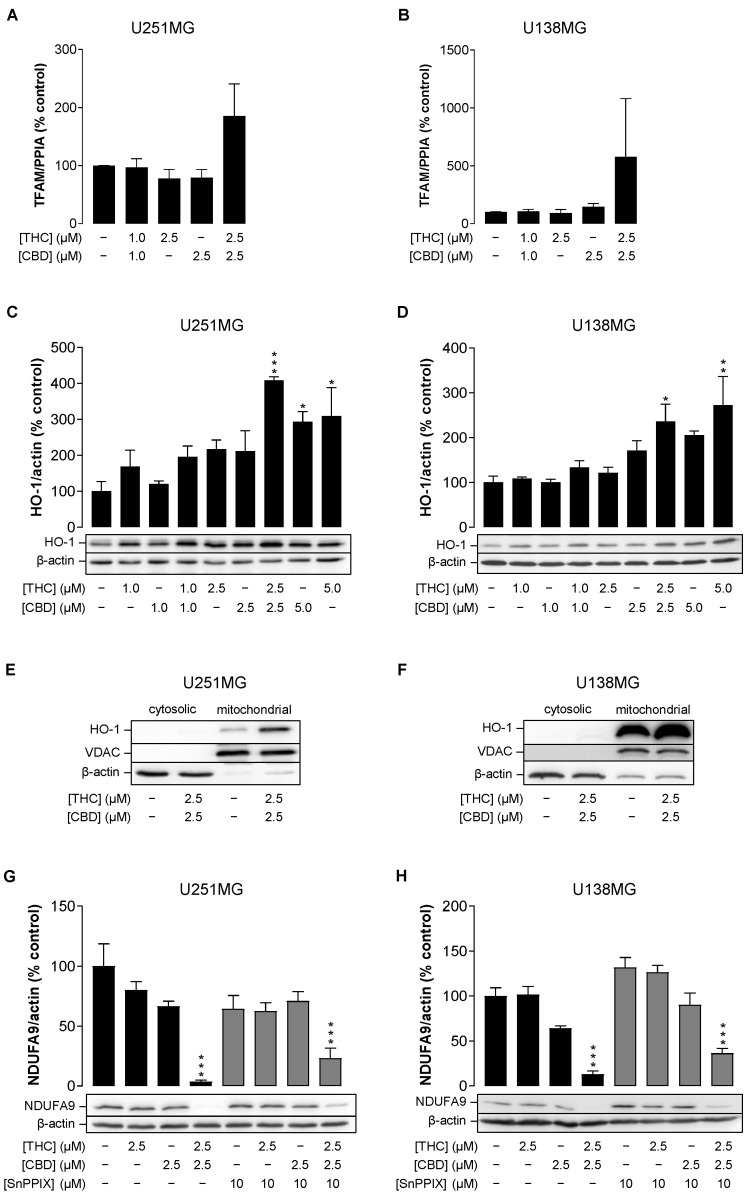
Influence of THC and CBD, administered alone or in combination, on mitochondrial transcription factor A (TFAM) expression (**A**,**B**) and heme oxygenase-1 (HO-1) protein levels (**C**–**F**) and function (**G**,**H**) in human glioblastoma cells. U251MG and U138MG cells were incubated with selected concentrations of THC and CBD, their combination (in a ratio of 1:1) or vehicle control for 24 h. In the experiments shown in Panels (**G**,**H**), the HO inhibitor SnPPIX (10 µM) was applied to the cells 1 h before the cannabinoids and was present throughout the incubation with the cannabinoids. Following incubation with cannabinoids, total RNA (**A**,**B**), cellular total protein (**C**,**D**,**G**,**H**) or mitochondrial fractions (**E**,**F**) were isolated and analysed for TFAM or HO-1. All percentage values shown refer to the vehicle control, which was set to 100%. The values given in the bar charts are based on RT-PCR (**A**,**B**) or densitometric analyses of blots (**C**,**D**,**G**,**H**), with the gene of interest normalised to PPIA (**A**,**B**) and the proteins normalised to β-actin (**C**,**D**,**G**,**H**). The blots shown are representative. The data in panels (**A**–**D**,**G**,**H**) are mean values ± SEM of *n* = 3–5 independent experiments per group. * *p* ≤ 0.05, ** *p* ≤ 0.01, *** *p* ≤ 0.001 vs. corresponding vehicle control, one-way ANOVA with Dunnett’s post hoc test (**A**–**D**) or Bonferroni’s post hoc test (**G**,**H**).

Instead of induced protein degradation, cannabinoids may influence protein levels of ETC subunits by disrupting their expression and ETC complex assembly. Therefore, two known players in the mechanism of complex synthesis of ETC complexes were investigated, namely mitochondrial transcription factor A (TFAM) and heme oxygenase-1 (HO-1). Both have been described as factors that can act both stimulatory [35,36,37] and inhibitory [35,38,39,40] on mitochondrial biogenesis and ETC complex assembly. In the case of the investigated combination of THC and CBD, a highly variable TFAM upregulation (Figure 6A,B) but a strong and significant induction of HO-1 (Figure 6C,D) was found in both cell lines. As shown in Figure 6E,F, the HO-1 induction occurred also in the mitochondrial fraction. Furthermore, experiments with the HO inhibitor SnPPIX resulted in a partial reversal of the NDUFA9 downregulation induced by THC/CBD (Figure 6G,H). This indicates that HO-1 may function as part of a network which regulates ETC protein levels in response to THC/CBD.

## 4. Discussion

A number of different cannabinoids have shown antitumour effects in many preclinical studies when applied individually (for review see [1,2]). In the present study, the combination of THC and CBD showed a synergistic inhibitory effect on the viability of human glioblastoma cells at 5 µM each, and can therefore be considered as a promising add-on therapy for glioblastoma, which is in agreement with the studies of other authors [6,7,8,9,10,11]. Yet it is this synergistic effect that has remained largely unexplored. Our study here provides a contribution to the still unknown molecular mechanism of action and reveals new targets of the cannabinoids located in the mitochondria of cancer cells. It shows that THC and CBD act on proteins of the mitochondrial ETC and thereby impair oxidative phosphorylation, leading to a breakdown of energy metabolism and cell death.

Similar to what was shown before by Marcu et al. [6], the synergism of THC and CBD in our study revealed itself in its strong reduction of cancer cell viability by combining 5 µM of each of the two cannabinoids. As we demonstrate for the first time, the enhanced inhibitory effect extends to the level of mitochondrial function, where a detailed analysis revealed a synergistic reduction in respiration by the combination of THC/CBD at 2.5 µM each. Thus, we found that the severe impairment of energy metabolism is associated with impaired respiration and damaged mitochondria, as shown by electron micrographs. This mitochondrial damage was registered on the basis of various parameters (morphology, cytochrome c release and ETC protein levels) and already occurred at cannabinoid concentrations at which the viability of the cells was only moderately reduced.

Previous studies had reported similar effects, but employed either high concentrations exceeding 5 µM for the cannabinoids, applied them individually and/or for very different durations. For example, high concentrations of CBD were reported to induce acute mitochondrial damage leading to cell death [18,19,41,42,43] in various cell types. Short incubation times with low micromolar concentrations of CBD showed only a weak effect on respiration in different cancer cells [14]; treatment above 12 h with CBD at low micromolar concentration in contrast induced a reduction of oxidative phosphorylation in cancer cells [16]. This latter finding is similar to our observations on OCR in glioblastoma cells, where incubation with 2.5 µM THC or CBD alone slightly reduced OCR, while the combination of both substances enhanced the effect. However, the detailed examination of mitochondrial respiratory capacities using the Seahorse analyser revealed that only the drug combination was effective, not the individual substances. This could suggest that CBD and THC cause further changes in metabolic capacities at the level of the whole cell, the investigation of which, however, was beyond the scope of the present study. Instead, we focused on the synergistic effect of the THC/CBD combination on mitochondria. Here, after treatment of cells with THC/CBD, an abnormal mitochondrial morphology characterised by swelling, loss of cristae structure and loss of mitochondrial membrane integrity with release of cytochrome c was observed, which has been previously reported for high concentrations of CBD alone [43]. Overall, our study thus largely confirms the findings of other groups that have reported the effects of individual cannabinoids on mitochondrial morphology and respiration, and further demonstrates that the combination of the two most commonly studied cannabinoids, THC and CBD, can synergistically enhance mitochondrial damage.

In the present work, it was shown that certain subunits of the ETC complex almost completely disappeared after treatment with THC and CBD in concentrations that only moderately affected the viability of the cells. Since this specific reduction of certain subunits occurred without any change in the amount of other mitochondrial proteins tested, especially VDAC, it cannot be explained by a change in mitochondrial mass. Likewise, in the electron microscopic images, mitochondria were clearly present, although the proximity of phagosomes to mitochondria and the presence of mitochondria inside phagosomes suggested that autophagy was activated by THC/CBD. Autophagy can occur as part of a protective mechanism promoting mitochondrial function or as a cell-destructive pathway downstream of damage to mitochondria and disruption of energy metabolism [30,44]. Both responses have been reported for cannabinoids in cancer cells, while most studies indicate that the cell-destructive pathway is favoured [15,45,46,47,48]. As the autophagy inhibitor bafilomycin A_1_ [49] did not significantly reduce ETC levels upon THC/CBD treatment in our study, it appears likely that autophagy is activated by the cells as a general response to mitochondrial damage and the loss of ETC complex I in particular. This interpretation is supported by studies in which ETC complexes were pharmacologically or genetically impaired, and autophagy and apoptosis were increased [29,50,51].

The analysis of the different subunits of the four complexes (I–IV) of the ETC and ATP synthase revealed that the subunits of complex I, NDUFA9 and NDUFB8, and complex IV, COX2 and COX4, were particularly severely affected by the THC/CBD-induced protein loss. The absence of subunits of the ETC complexes, even those that only have a stabilising function and are not directly involved in electron transport, is sufficient to massively disrupt the functionality of the entire complex I, and could thus explain the loss of OCR in our study [52,53]. For example, NDUFA9, which is responsible for stabilising the junction between the membrane and matrix arms of complex I [54], has been shown to be strictly required for complex I assembly and function [52]. This opens two general pathways for how NDUFA9 levels could regulate complex I stability: either NDUFA9 is degraded and complex I disassembled upon THC/CBD exposure, or complex I fails to assemble properly due to a dysregulation of structural assembly factors, of which NDUFA9 is an integral component.

In investigating possible triggers for the loss of complex I, it was first examined whether the effects of THC and CBD were mediated by their classical cannabinoid receptors at the plasma membrane and in the mitochondria [55,56]. To address this question, the decrease in NDUFA9 was measured in the presence of the THC/CBD combination and antagonists for CB_1_, CB_2_ and TRPV1. Notably, this approach showed that none of these receptors of the classical cannabinoid response cascade caused the observed decrease in NDUFA9, which appears to be in contrast to reports in the literature. For example, Kim et al. reported that TRPV1 is responsible for triggering mitochondrial damage in microglial cells, which ultimately led to apoptosis [57]. Similarly, Fišar et al. have demonstrated a dependence of THC-induced apoptosis on CB_1_ receptor-mediated damage to mitochondria isolated from pig brains [58]. Alternatively, THC/CBD could reduce NDUFA9 levels in the glioblastoma cells through other mechanisms. Accordingly, a number of non-classical receptors to which THC and CBD bind have been discovered in recent years [1,48]. Furthermore, a direct interaction between CBD and the outer mitochondrial membrane channel VDAC was discovered [18,41], and a receptor-independent effect of CBD on mitochondria in leukaemia cells was observed [43]. All these interactions should still be tested for their role in the regulation of ETC proteins and mitochondrial function. In addition, the antagonist/inverse agonist of CB_1_ receptors AM-251 reduced NDUFA9 in the absence of phytocannabinoids in our study. Fišar et al. [58] reported that AM-251 alone reduced respiration by inhibiting complex I and II in isolated mitochondria, which was significantly attributed to non-receptor-associated effects of the compound. Similarly, AM-251 reduced mitochondrial bioenergetic functions as a result of off-target effects in human pancreatic cancer cells [59]. Whether similar off-target effects occur in the glioblastoma cells and whether these specifically affect NDUFA9 levels remain to be investigated.

There are several known pathways which regulate the turnover of NDUFA9. The proteasome is a cytosolic machinery responsible for the degradation of redundant and damaged proteins mainly in the cytosol, but it is also involved in mitochondrial protein quality control [60]. In this context, the proteasome typically acts on mitochondrial precursor proteins as well as outer membrane proteins, whereas an activity on inner membrane proteins has so far only been demonstrated for SDHA [60]. The effective proteasome inhibitors bortezomib and MG-132 failed to reverse the loss of NDUFA9 in our study; therefore, it seems unlikely that the proteasome is involved in the NDUFA9 reduction induced by the combination of THC and CBD.

Mitochondrial proteins are also degraded constitutively as part of a quality control mechanism by mitochondrial proteases, a large protein family with mostly low target specificity [34]. Thus, the main function of the mitochondrial protease ClpXP is thought to be the degradation of misfolded proteins, while LONP is responsible for the removal of unassembled proteins or proteins with mild oxidative damage [34]. Both LONP and ClpXP are also activated in response to stress [34,61]. Since we could neither detect a cannabinoid-induced upregulation of LONP nor was the LONP inhibitor MG-132 [33] able to rescue NDUFA9, an involvement of this mitochondrial protease in the THC/CBD-mediated loss of NDUFA9 seems very unlikely. However, mitochondria express several other proteases that have not yet been fully characterised, so we cannot rule out the possibility that one or more of them are involved in THC/CBD-mediated NDUFA9 turnover.

As proteases and the proteasome constitute the main pathways for the degradation of mitochondrial proteins, and neither is likely to contribute in a major role to THC/CBD-induced loss of NDUFA9, it is also possible that THC/CBD affects NDUFA9 levels mostly as part of a larger network of assembly and stabilising factors for complex I. For the assembly of the ETC complexes, all subunits need to be present and interact with many other assembly factors in an intricate network [62,63]. NDUFA9 is produced in large quantities and degraded if the subunits and folding factors of assembly are absent [64]. Hence, instead of an increased degradation of the protein, a disturbed production of important subunits and thus a defective assembly of the complexes could also explain our findings.

Such larger regulatory networks tend to be controlled by a common factor. As a matter of fact, HO-1 is an important regulator of mitochondrial biogenesis and ETC synthesis, as it plays a central role in iron homeostasis in mitochondria and regulates several factors in mitochondrial biogenesis, including TFAM [37,65,66,67]. In cancer cells, HO-1 plays a dual role in cell protection and cell damage [68]. As recently shown, depending on the magnitude of induction, CBD-induced expression of this enzyme by stress can be cytoprotective, but can also lead to cell death [69]. The strong upregulation of HO-1 especially in mitochondria and the partial restoration of NDUFA9 by SnPPIX after THC/CBD treatment of glioblastoma cells demonstrated in the present study suggests that HO-1 may contribute to protein loss to some extent. In line with this assumption, a similar destructive response to HO-1 elevation specifically in mitochondria was reported in cells exposed to hypoxia [39]. However, the only partially protective effect shown in the SnPPIX experiment also suggests that HO-1 may only be part of a broader regulatory network to reduce ETC protein levels that responds to THC/CBD and that remains to be characterised.

Aside from the expression of known regulators of mitochondrial biogenesis, there are reports associating CBD-induced imbalance of mitochondrial expression with mitochondrial function, e.g., in hepatocellular carcinoma cells [19]. CBD was also found to reduce prohibitin, a crucial protein for mitochondrial dynamics and biogenesis, in various cancer cells [14]. Similarly, heat shock proteins are linked to mitochondrial biogenesis [70,71]. It is possible that the THC/CBD-induced reduction of NDUFA9 is caused by altered production of such accessory factors to complex formation rather than NDUFA9 itself, but this remains to elucidated.

## 5. Conclusions

The combination of THC and CBD represents an interesting new option for an add-on therapeutic strategy for the treatment of gliomas. However, the molecular mechanism of the synergistic effect exerted by the two phytocannabinoids in cancer cells is still unknown. The present study revealed new insights into the effect mediated by the combination of THC and CBD: cannabinoids reduce the levels of specific subunits of complexes I and IV, and their depletion leads to reduced mitochondrial respiration and induction of autophagy and apoptosis in glioma cells. Although understanding the mechanism in full requires further investigation, our study points to a promising new direction for the development of cancer therapy strategies.

## Data Availability

Data are available upon reasonable request from the first author.

## Supplementary Materials

The following supporting information can be downloaded at: https://www.mdpi.com/article/10.3390/cancers14133129/s1, Figure S1: Influence of THC and CBD, administered alone or in combination, on lactate secretion; Figure S2: Role of the proteasome in downregulation of the mitochondrial subunit NDUFA9 by the combination of THC and CBD in human glioblastoma cells; Figure S3: Influence of THC and CBD, administered alone or in combination, on LONP levels in human glioblastoma cells. File S1: Uncropped Western blot figures.
