# Supplementary material for: The Combination of Δ9-Tetrahydrocannabinol and Cannabidiol Suppresses Mitochondrial Respiration of Human Glioblastoma Cells via Downregulation of Specific Respiratory Chain Proteins
Source: Cancers (Basel). 2022 Jun 27;14(13):3129. doi: 10.3390/cancers14133129 (PMC9265124; doi:10.3390/cancers14133129)
Supplement: Supplementary file 1 [file cancers-14-03129-s001.zip › cancers-1713414 - File S1 - Uncropped WB blots.pdf]

Fig. 1G

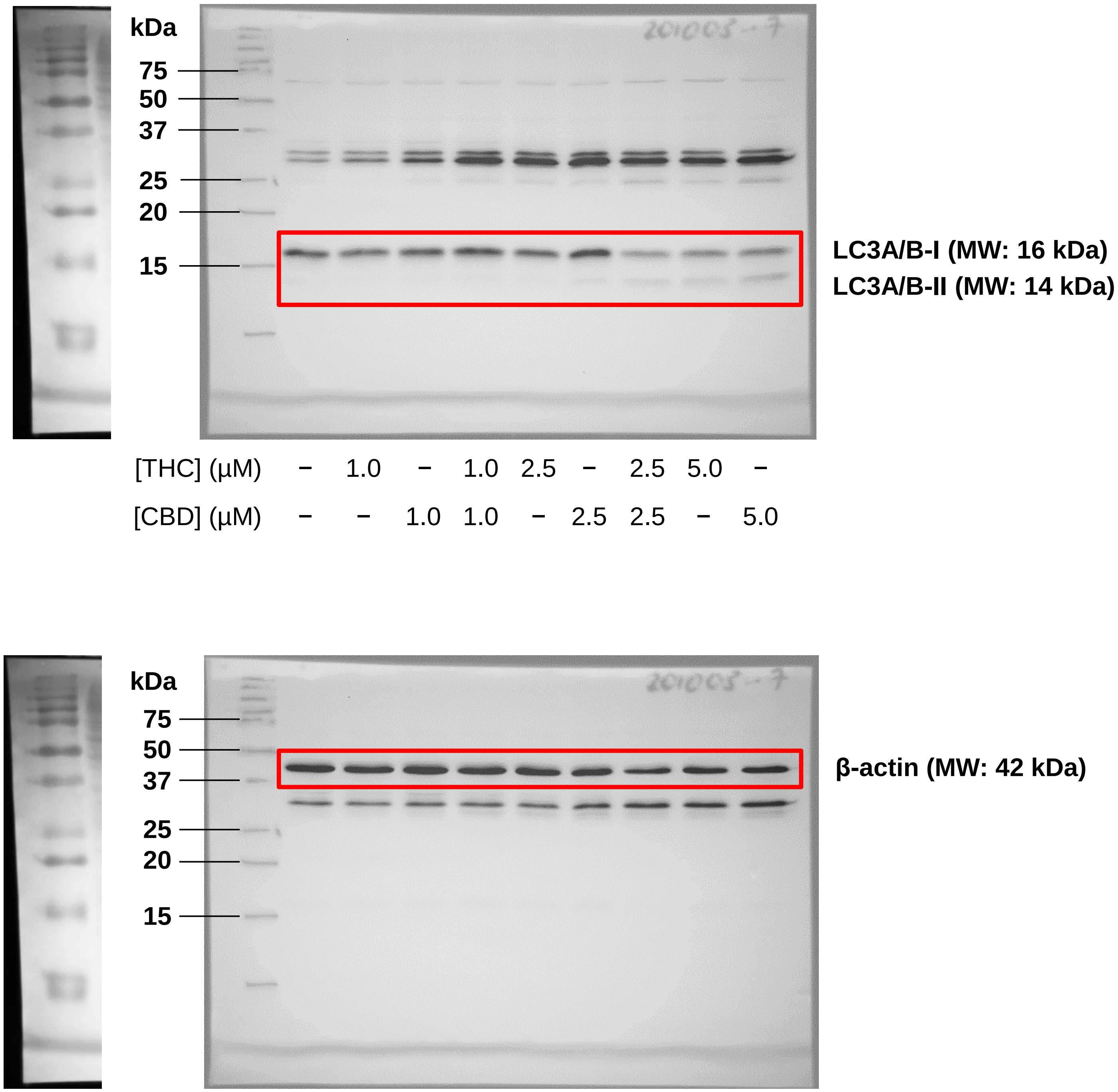
kDa
75
50
37
25
20
15
LC3A/B-I (MW: 16 kDa)
LC3A/B-II (MW: 14 kDa)
[THC] (μM) – 1.0 – 1.0 2.5 – 2.5 5.0 –
[CBD] (μM) – – 1.0 1.0 – 2.5 2.5 – 5.0
kDa
75
50
37
25
20
15
β-actin (MW: 42 kDa)

Fig. 1H

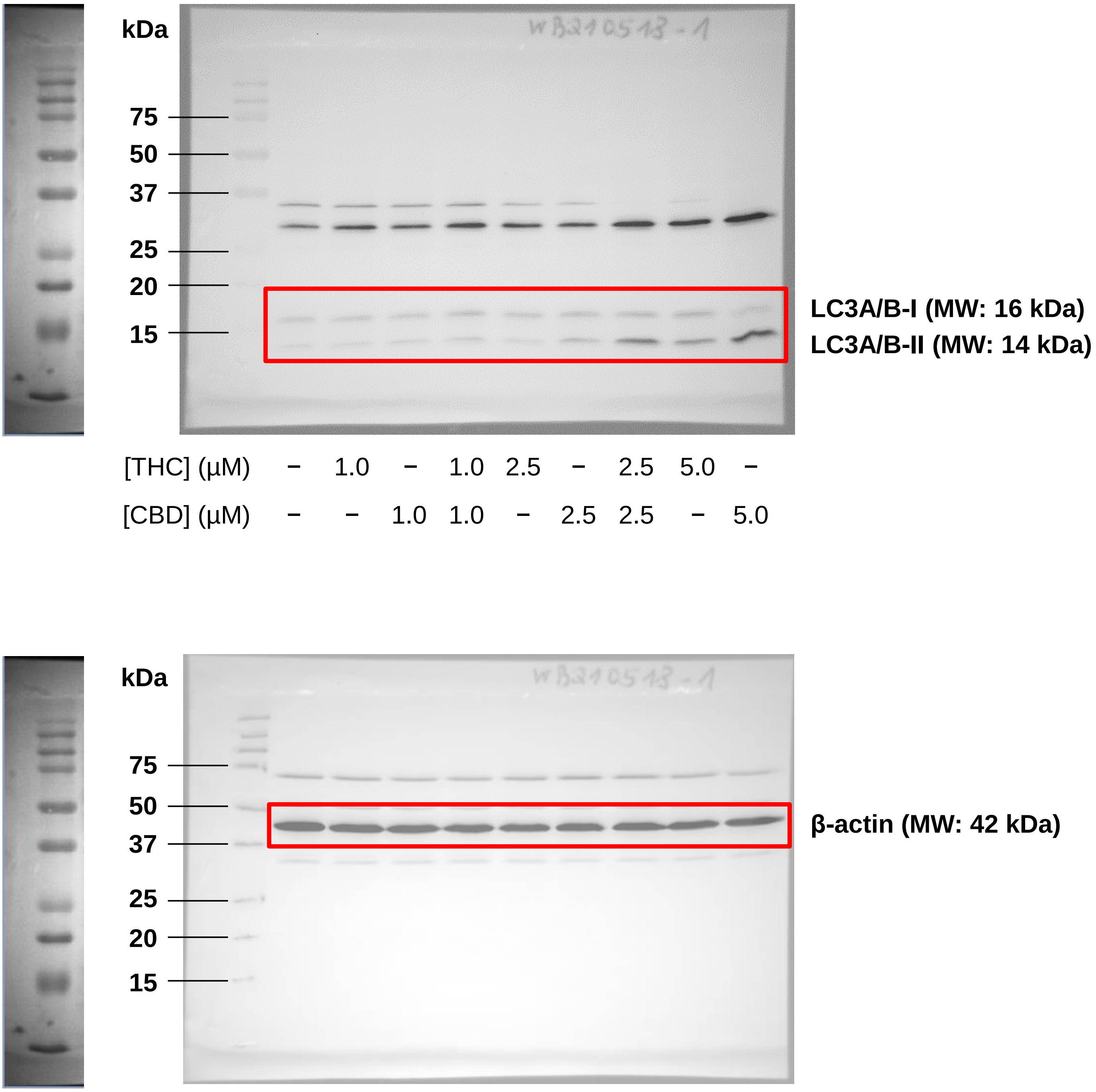
kDa
75
50
37
25
20
15
LC3A/B-I (MW: 16 kDa)
LC3A/B-II (MW: 14 kDa)
[THC] (μM) – 1.0 – 1.0 2.5 – 2.5 5.0 –
[CBD] (μM) – – 1.0 1.0 – 2.5 2.5 – 5.0
kDa
75
50
37
25
20
15
β-actin (MW: 42 kDa)

Fig. 3G left side

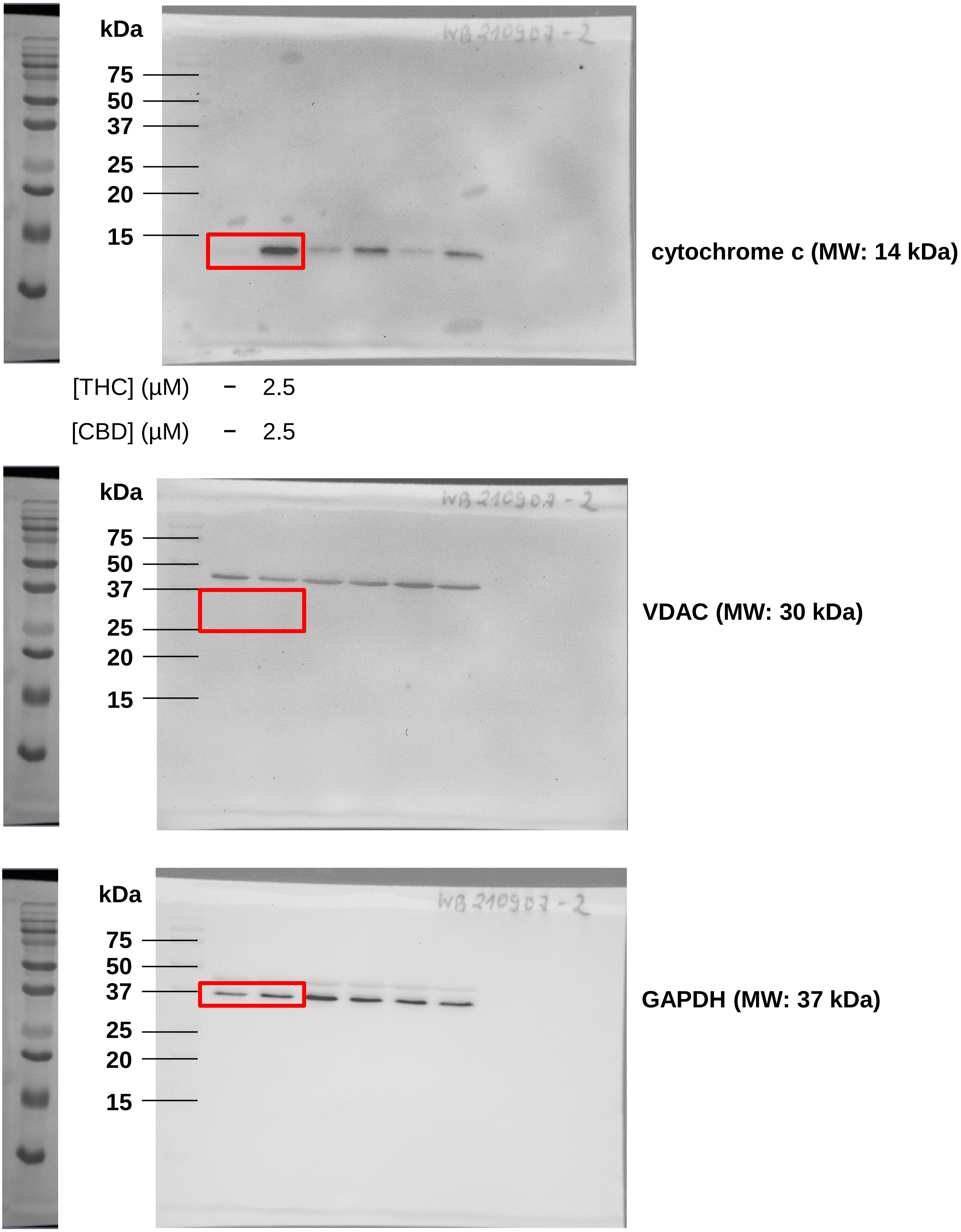

Fig. 3G right side

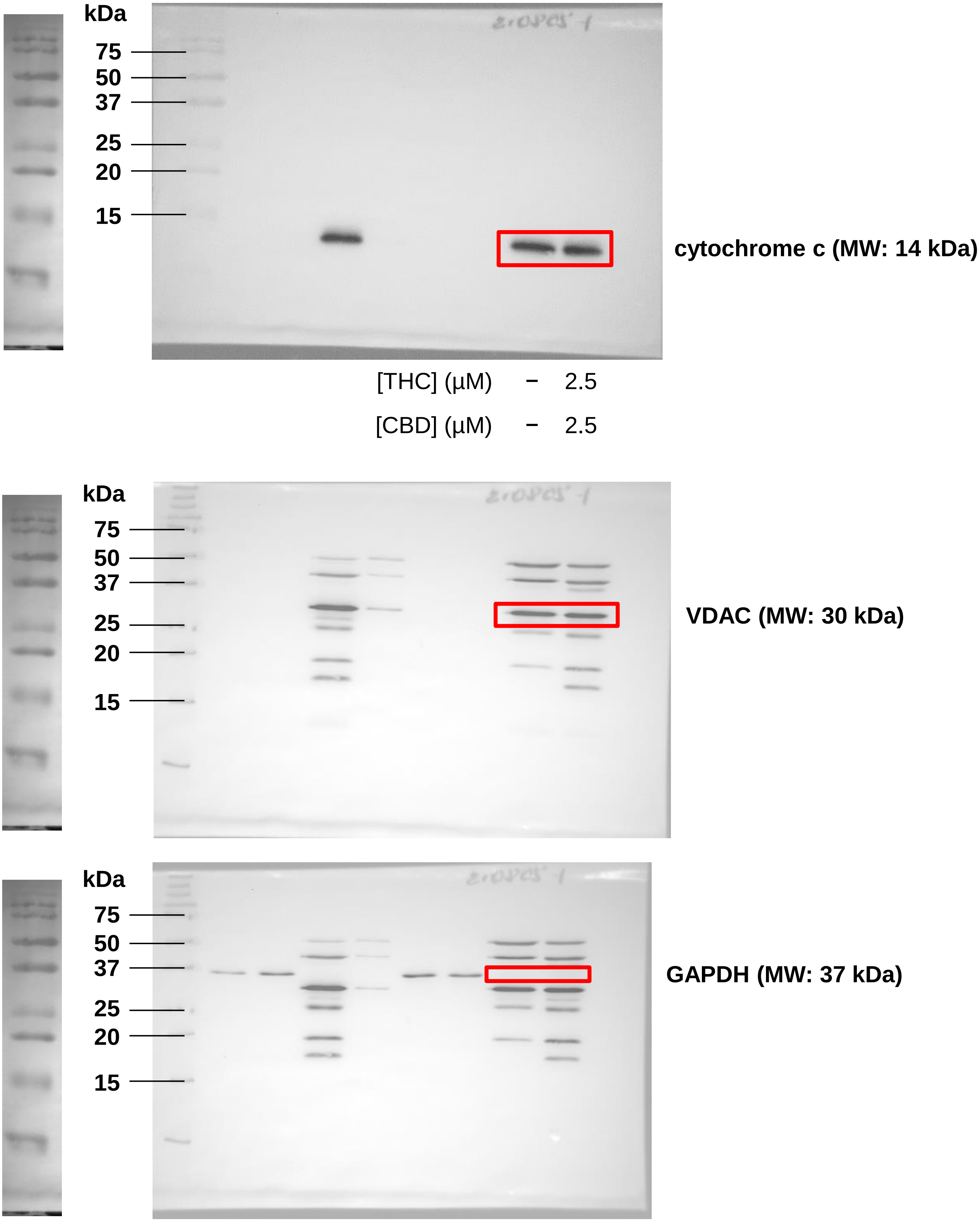

Fig. 3H left side

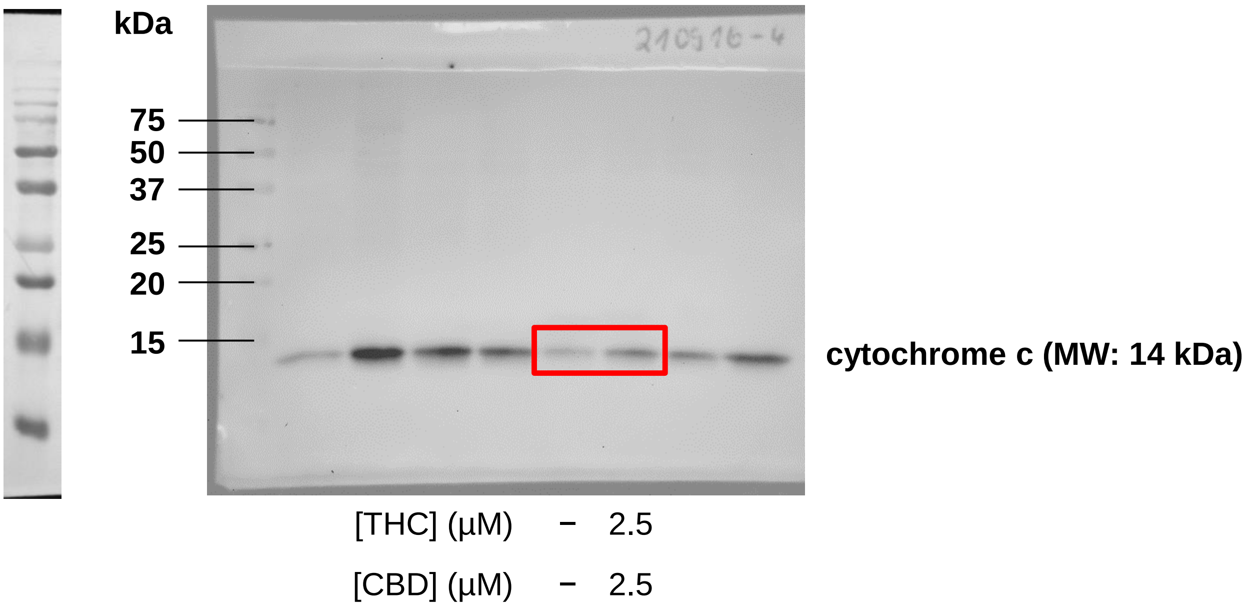

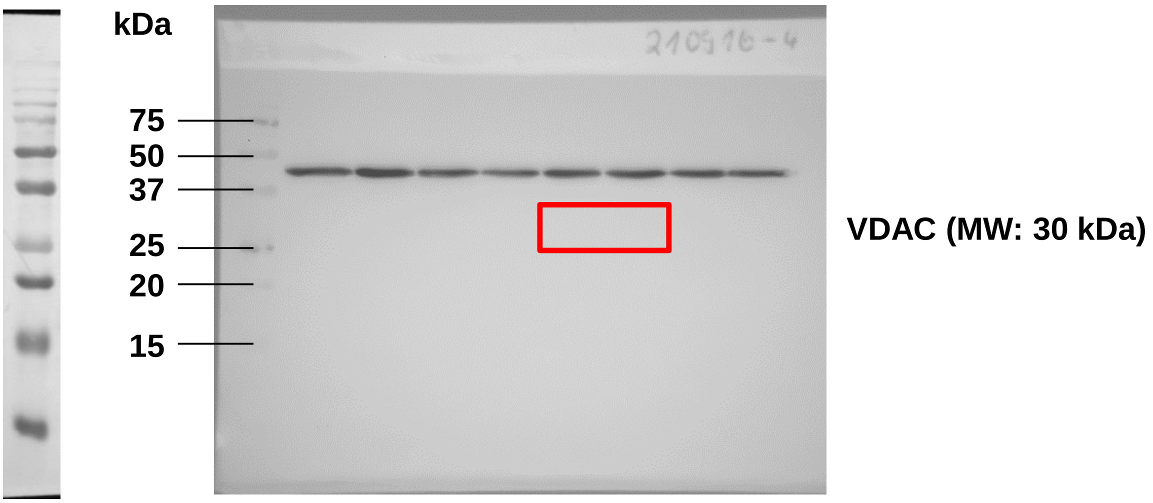

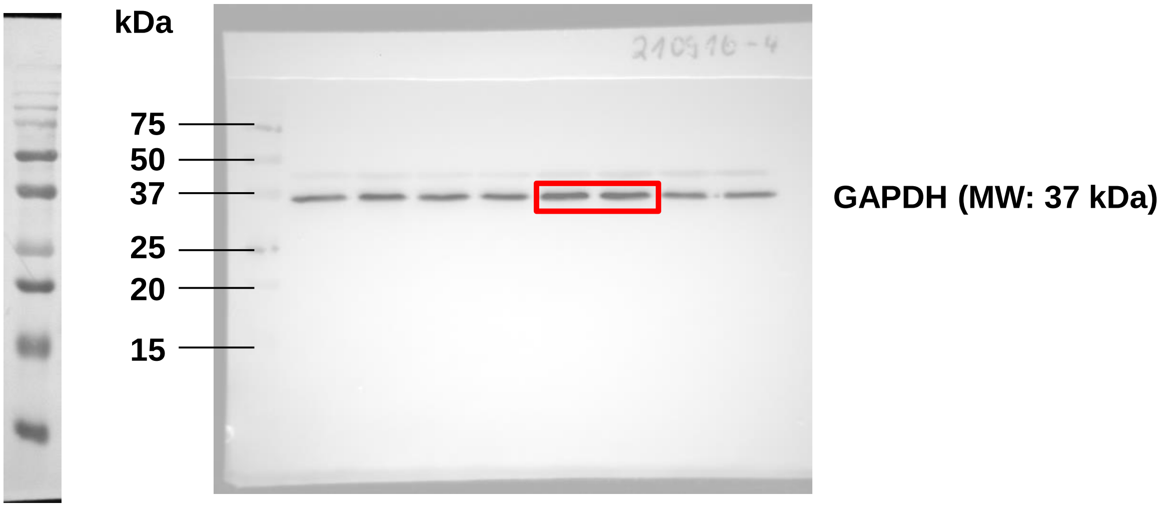

Fig. 3H right side

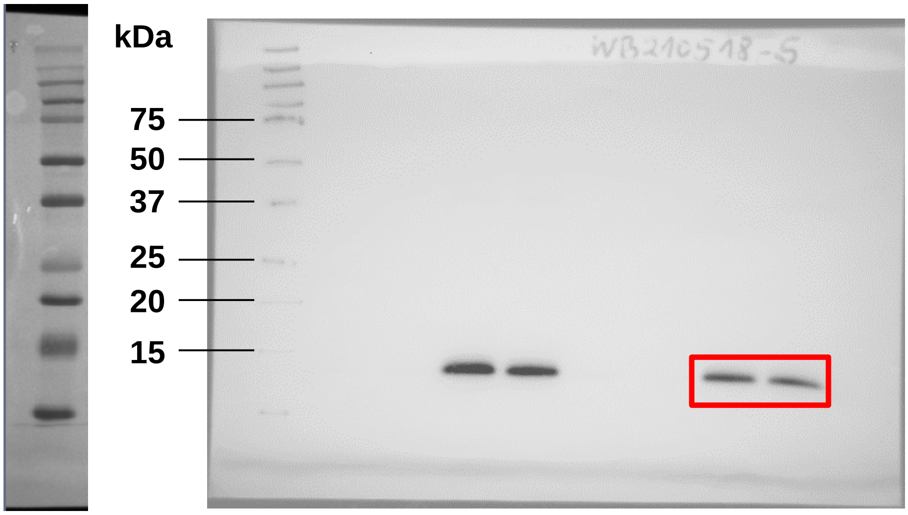

cytochrome c (MW: 14 kDa)

[THC] (µM)	–	2.5
[CBD] (µM)	–	2.5

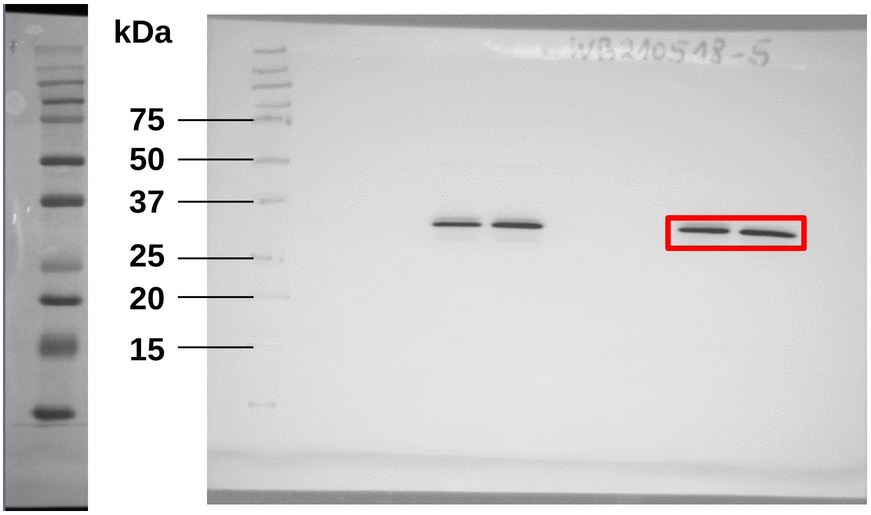

VDAC (MW: 30 kDa)

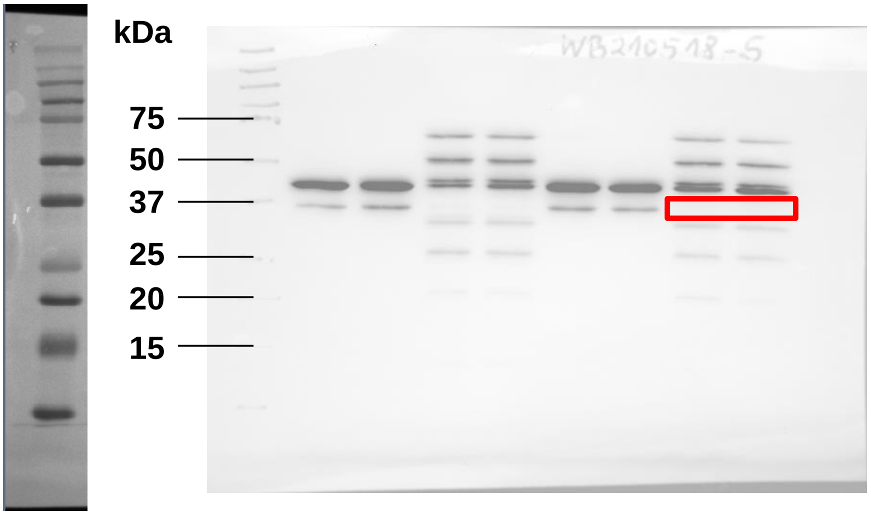

GAPDH (MW: 37 kDa)

Fig. 4A

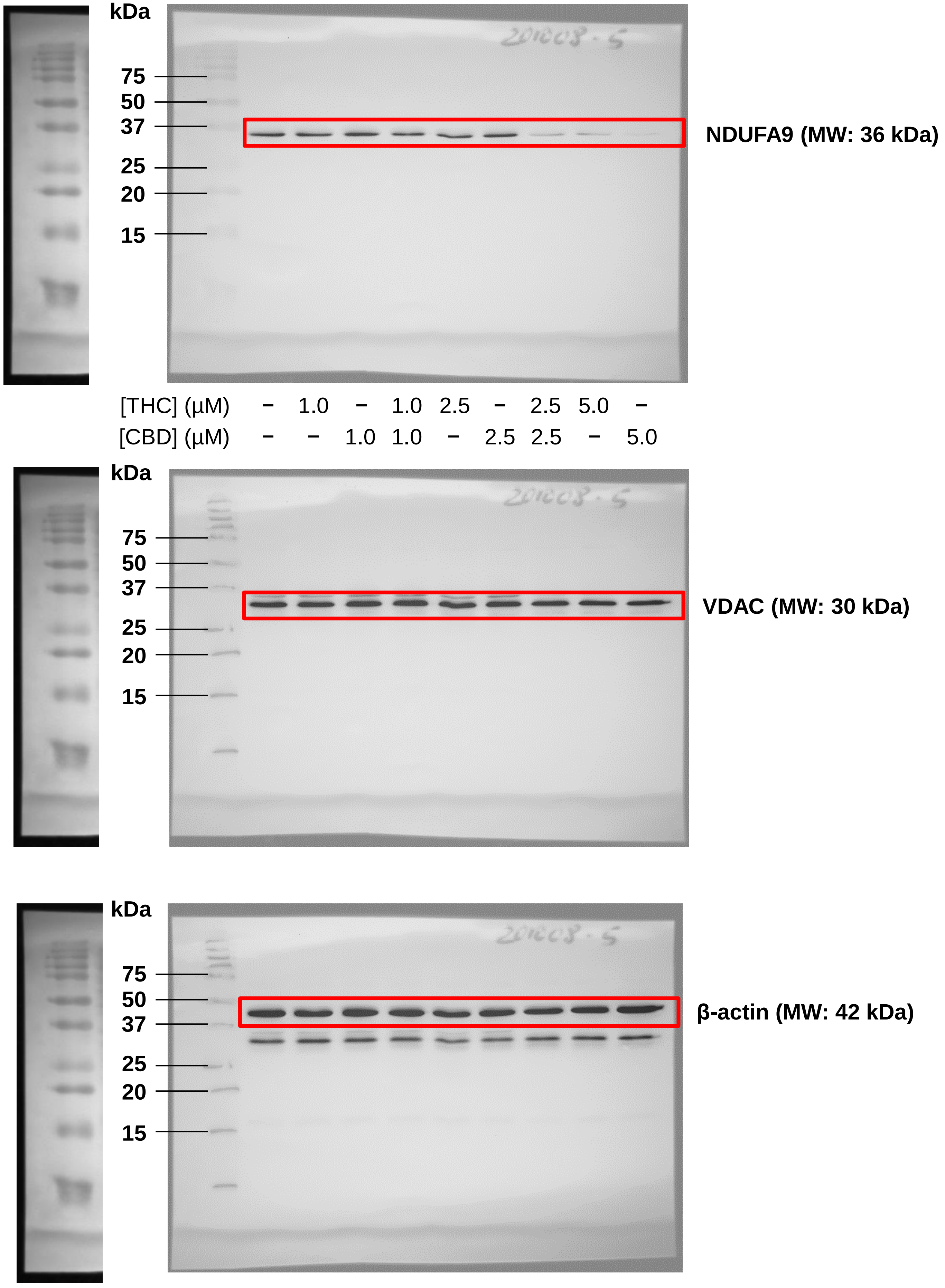
kDa
75
50
37
25
20
15
NDUFA9 (MW: 36 kDa)
[THC] (µM) – 1.0 – 1.0 2.5 – 2.5 5.0 –
[CBD] (µM) – – 1.0 1.0 – 2.5 2.5 – 5.0
kDa
75
50
37
25
20
15
VDAC (MW: 30 kDa)
kDa
75
50
37
25
20
15
β-actin (MW: 42 kDa)

Fig. 4A cont.

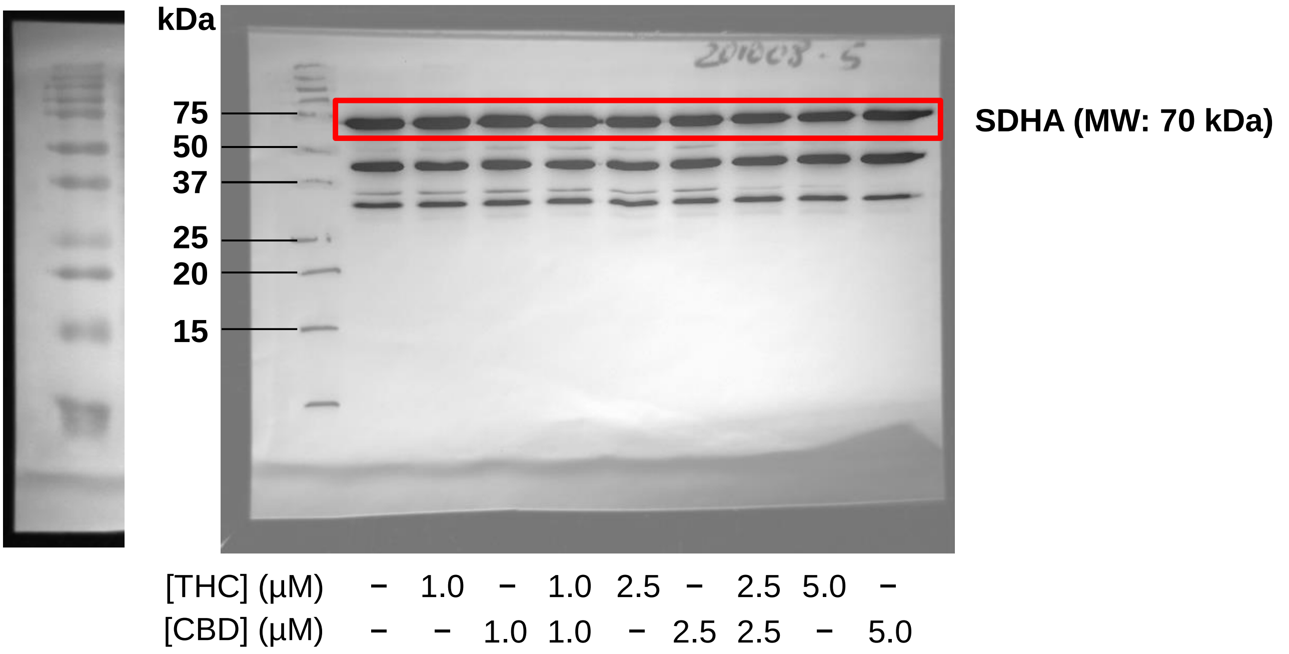

kDa
75
50
37
25
20
15
SDHA (MW: 70 kDa)
[THC] (µM) – 1.0 – 1.0 2.5 – 2.5 5.0 –
[CBD] (µM) – – 1.0 1.0 – 2.5 2.5 – 5.0

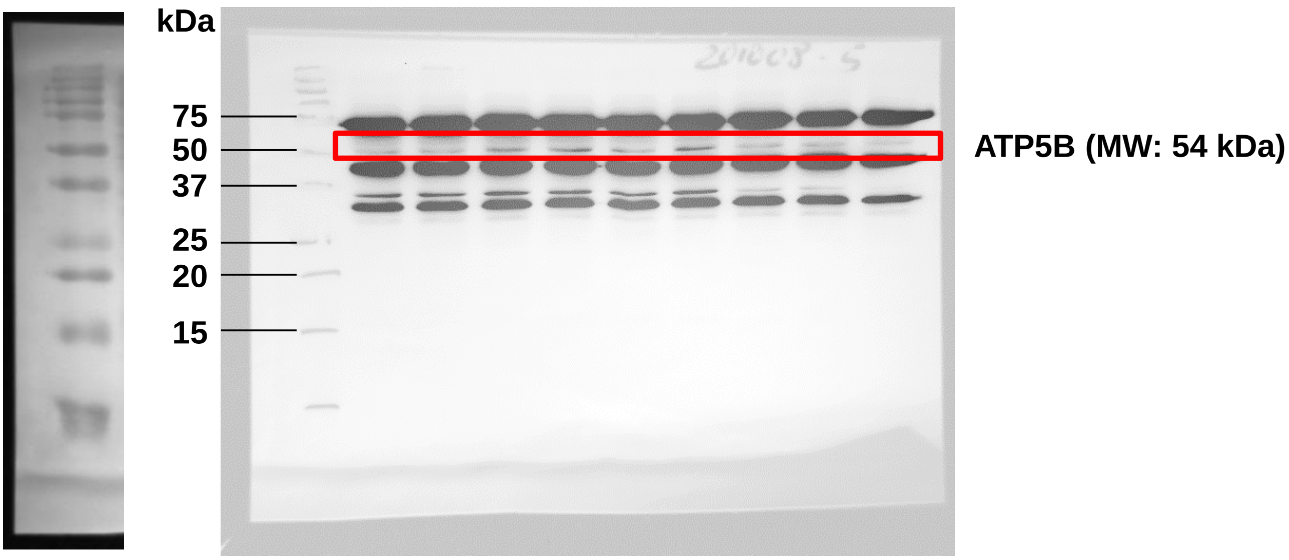

kDa
75
50
37
25
20
15
ATP5B (MW: 54 kDa)

Fig. 4B

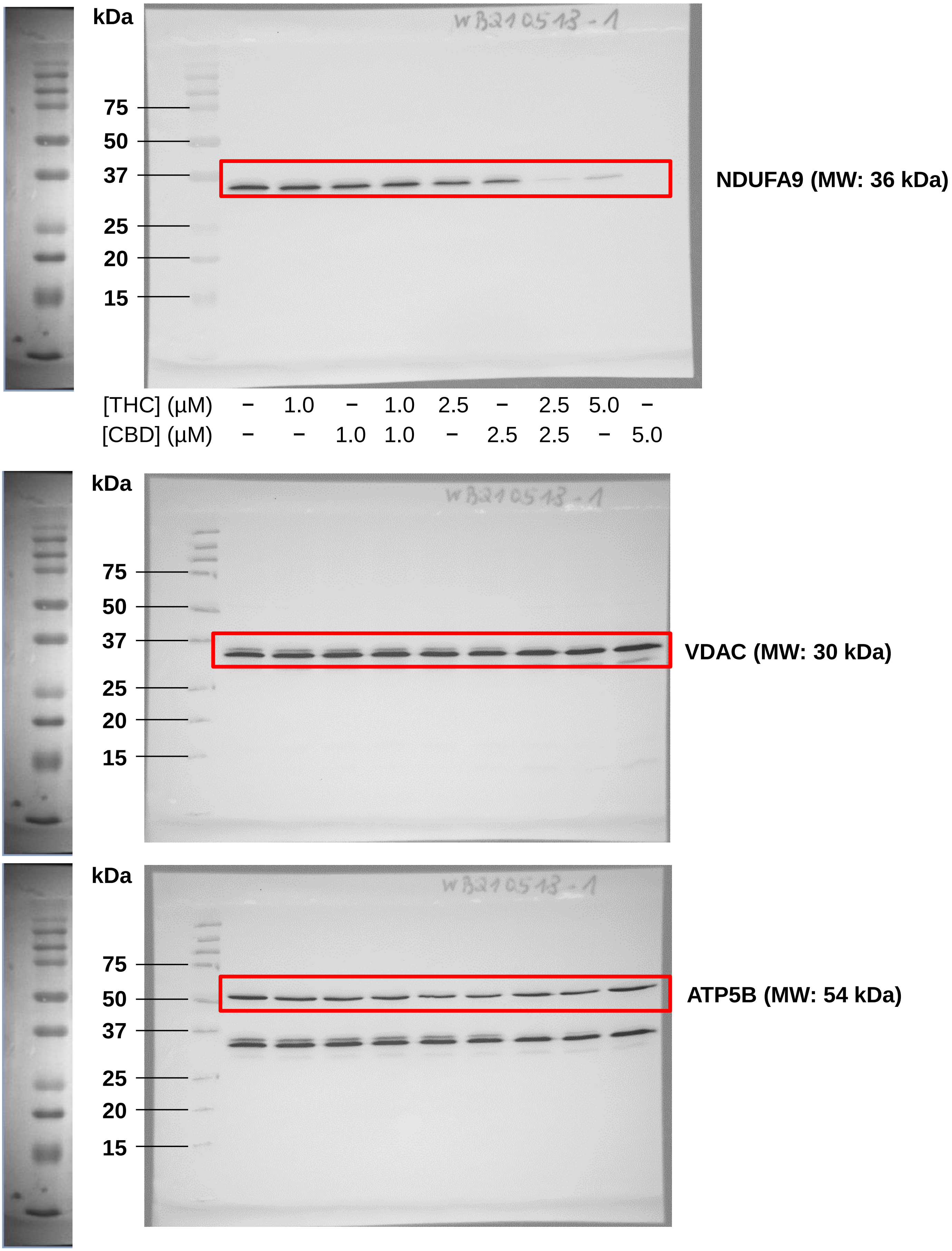
kDa
75
50
37
25
20
15
NDUFA9 (MW: 36 kDa)
[THC] (μM) – 1.0 – 1.0 2.5 – 2.5 5.0 –
[CBD] (μM) – – 1.0 1.0 – 2.5 2.5 – 5.0
kDa
75
50
37
25
20
15
VDAC (MW: 30 kDa)
kDa
75
50
37
25
20
15
ATP5B (MW: 54 kDa)

Fig. 4B cont.

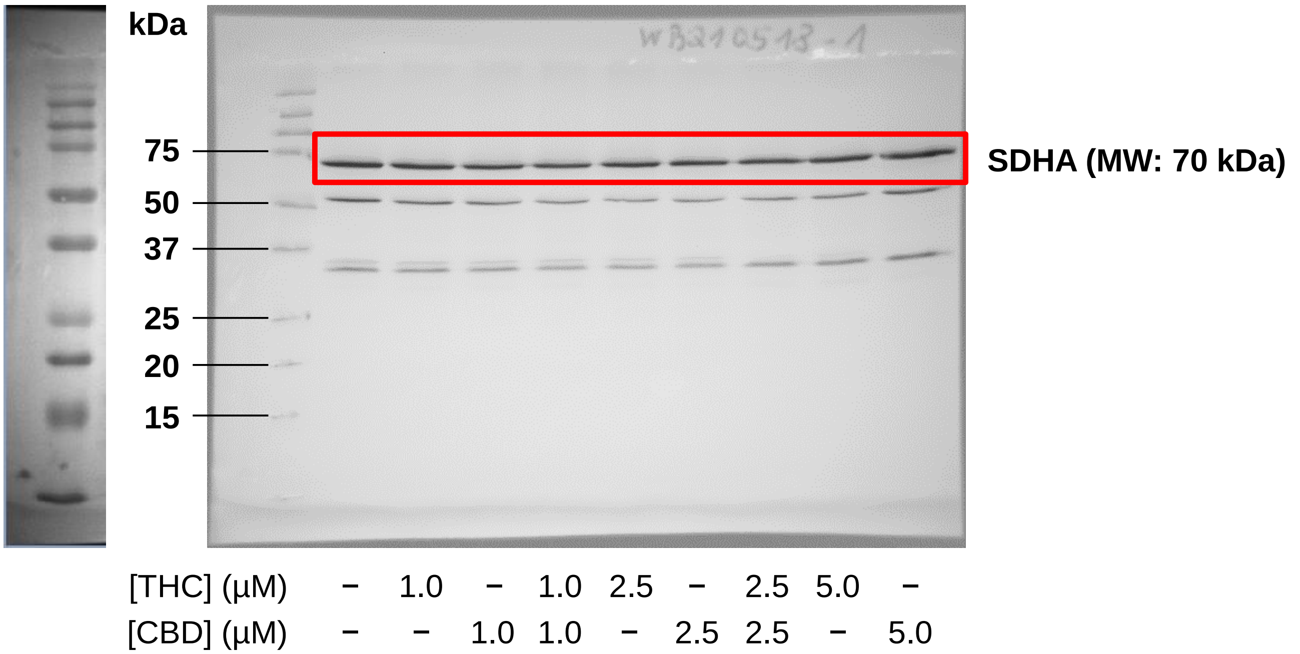
kDa
75
50
37
25
20
15
SDHA (MW: 70 kDa)
[THC] (µM) – 1.0 – 1.0 2.5 – 2.5 5.0 –
[CBD] (µM) – – 1.0 1.0 – 2.5 2.5 – 5.0

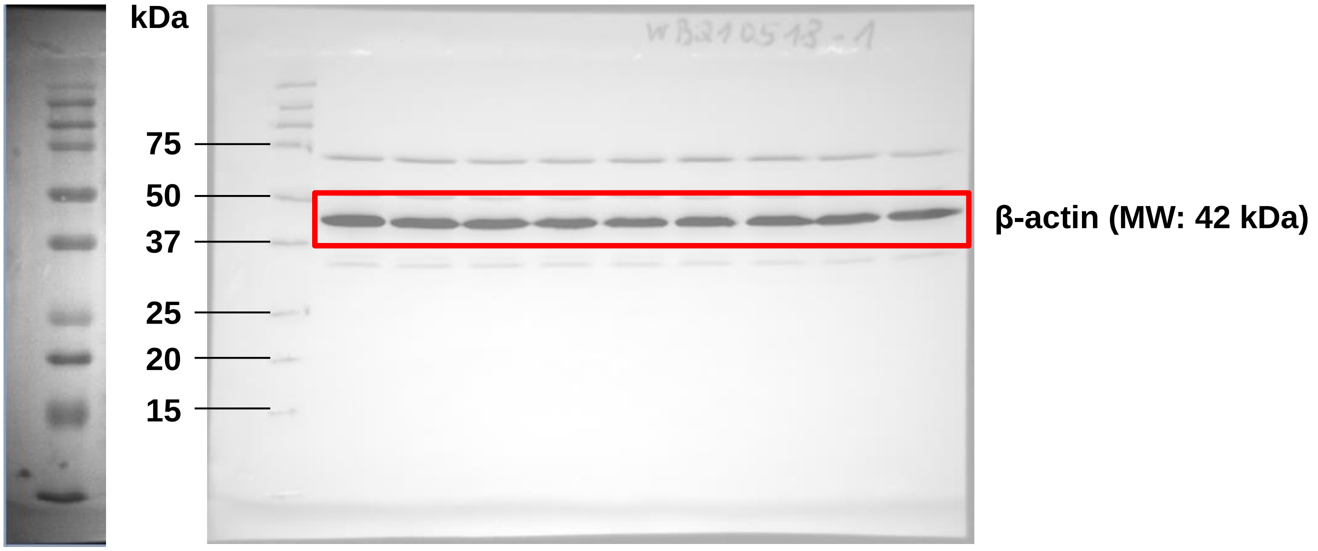
kDa
75
50
37
25
20
15
β-actin (MW: 42 kDa)

Fig. 4C

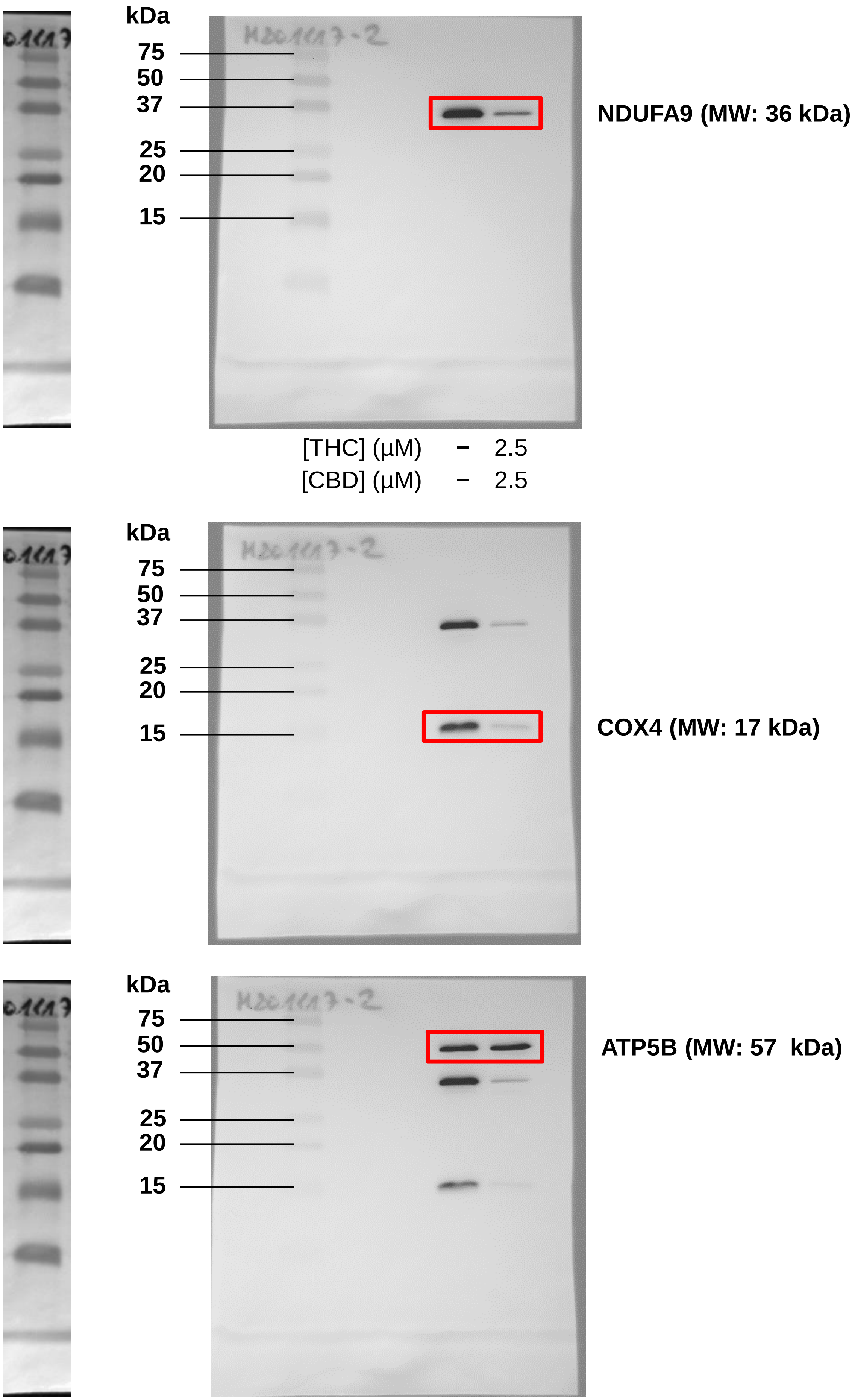
kDa
75
50
37
25
20
15
NDUFA9 (MW: 36 kDa)
[THC] (μM) – 2.5
[CBD] (μM) – 2.5
kDa
75
50
37
25
20
15
COX4 (MW: 17 kDa)
kDa
75
50
37
25
20
15
ATP5B (MW: 57 kDa)

Fig. 4C cont.

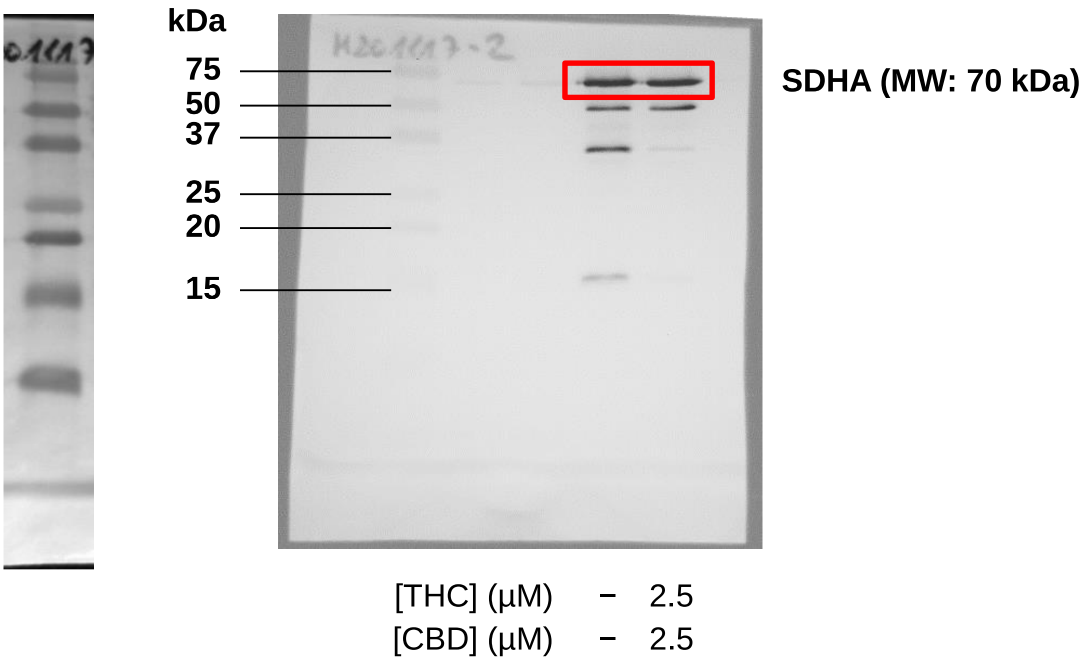

kDa
75
50
37
25
20
15
SDHA (MW: 70 kDa)
[THC] (µM) – 2.5
[CBD] (µM) – 2.5

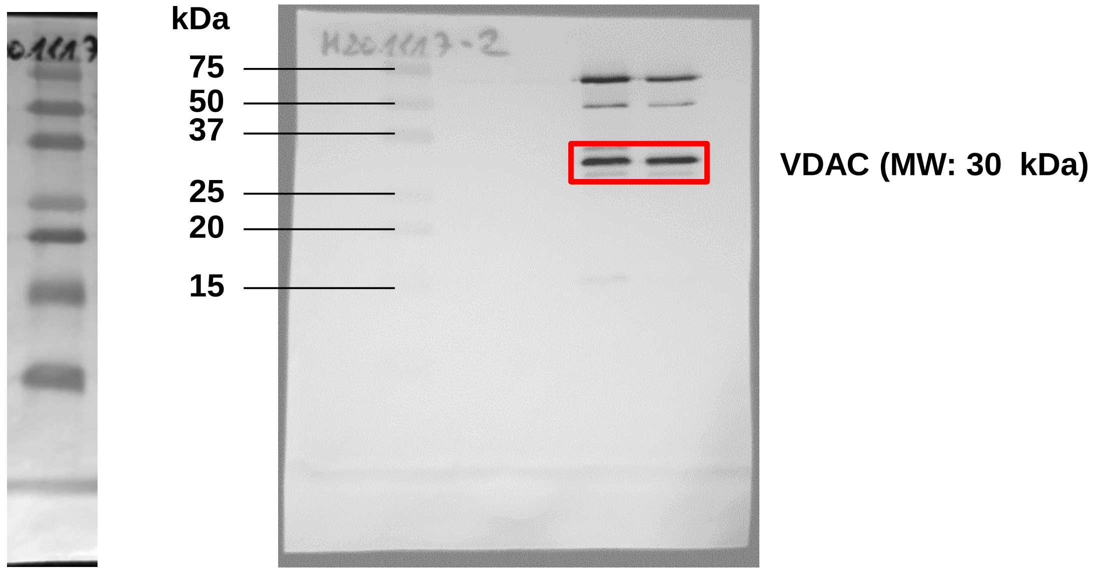

kDa
75
50
37
25
20
15
VDAC (MW: 30 kDa)

Fig. 4D

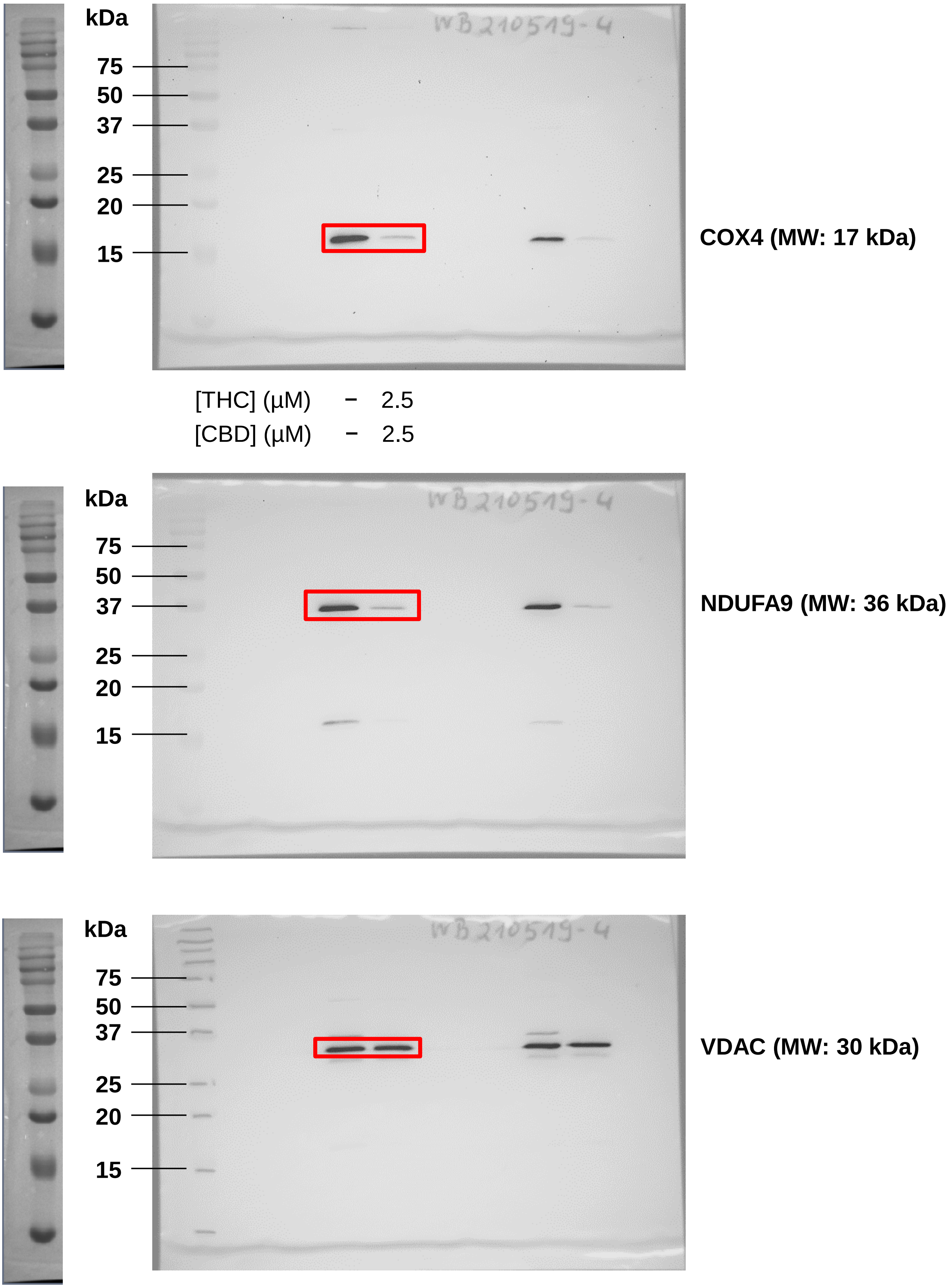
kDa
75
50
37
25
20
15
COX4 (MW: 17 kDa)
[THC] (µM) – 2.5
[CBD] (µM) – 2.5
kDa
75
50
37
25
20
15
NDUFA9 (MW: 36 kDa)
kDa
75
50
37
25
20
15
VDAC (MW: 30 kDa)

Fig. 4D cont.

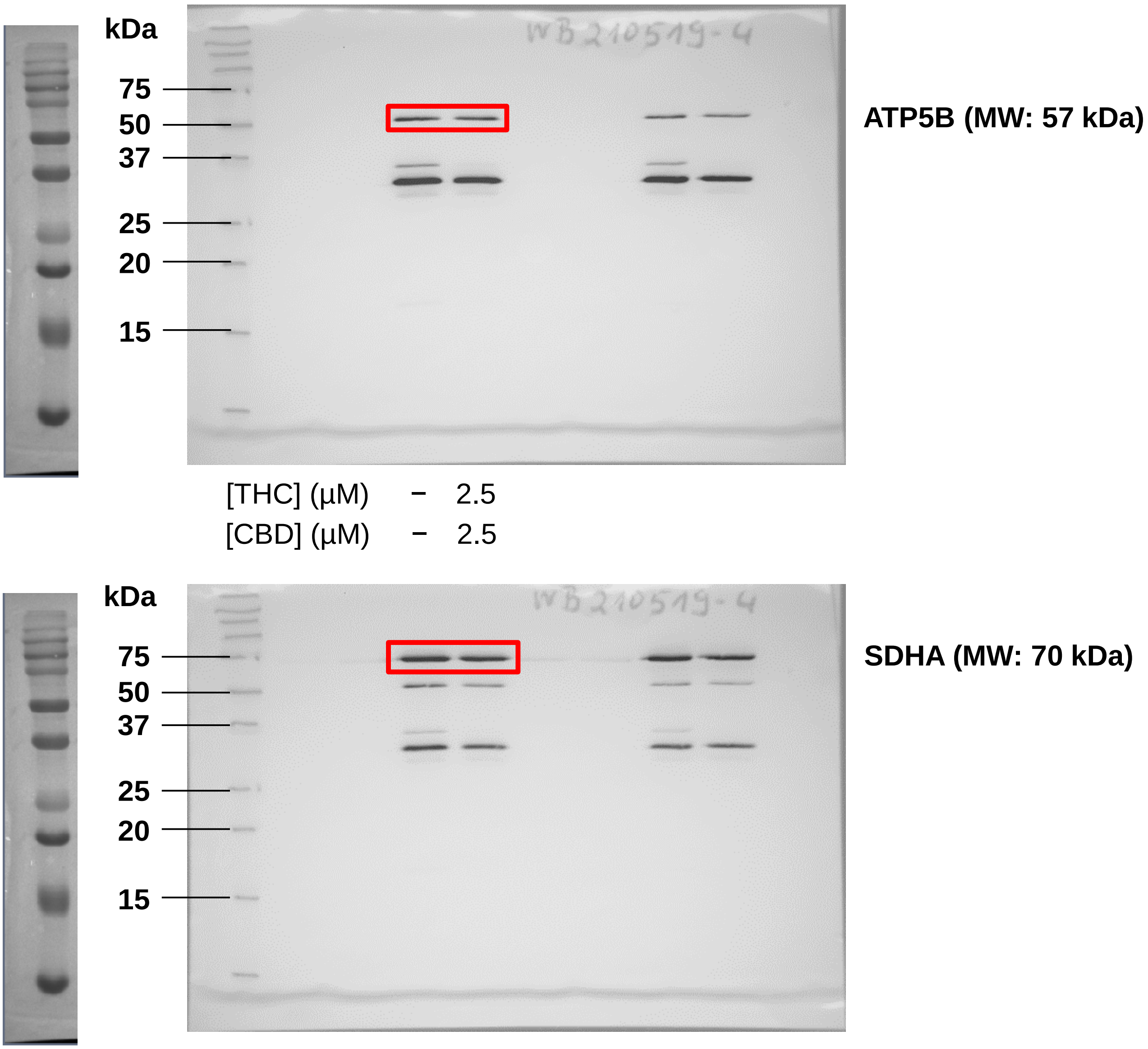
kDa
75
50
37
25
20
15
ATP5B (MW: 57 kDa)
[THC] (µM) − 2.5
[CBD] (µM) − 2.5
kDa
75
50
37
25
20
15
SDHA (MW: 70 kDa)

Fig. 4E

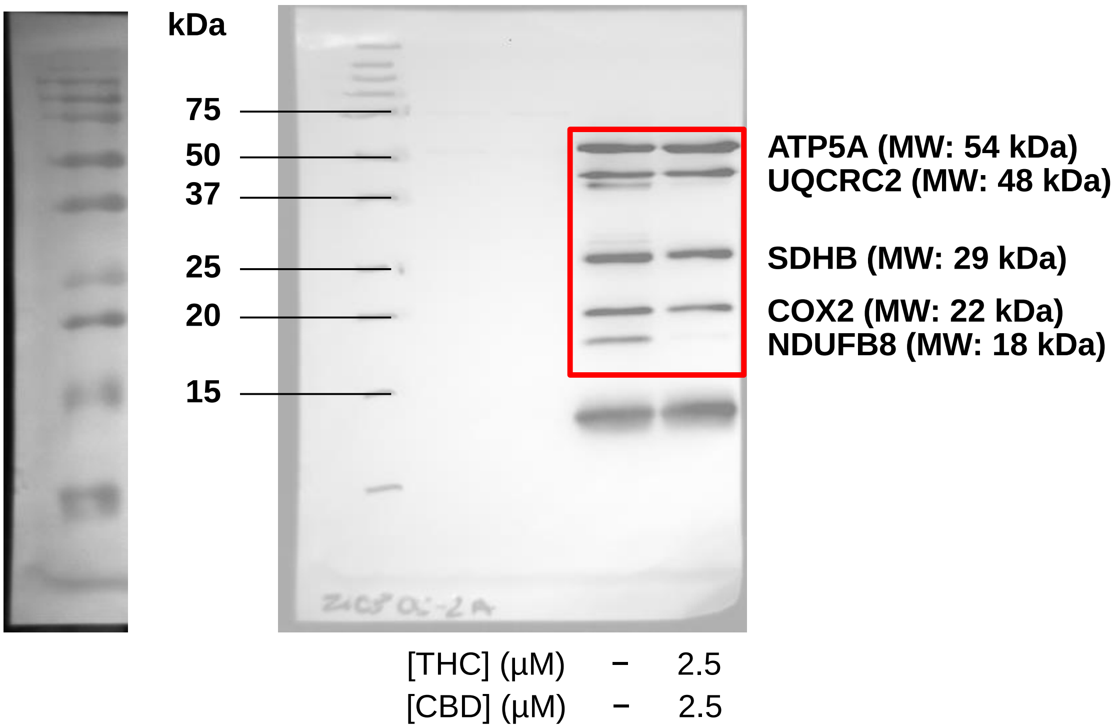
kDa
75
50
37
25
20
15
ATP5A (MW: 54 kDa)
UQCRC2 (MW: 48 kDa)
SDHB (MW: 29 kDa)
COX2 (MW: 22 kDa)
NDUFB8 (MW: 18 kDa)
[THC] (μM) – 2.5
[CBD] (μM) – 2.5

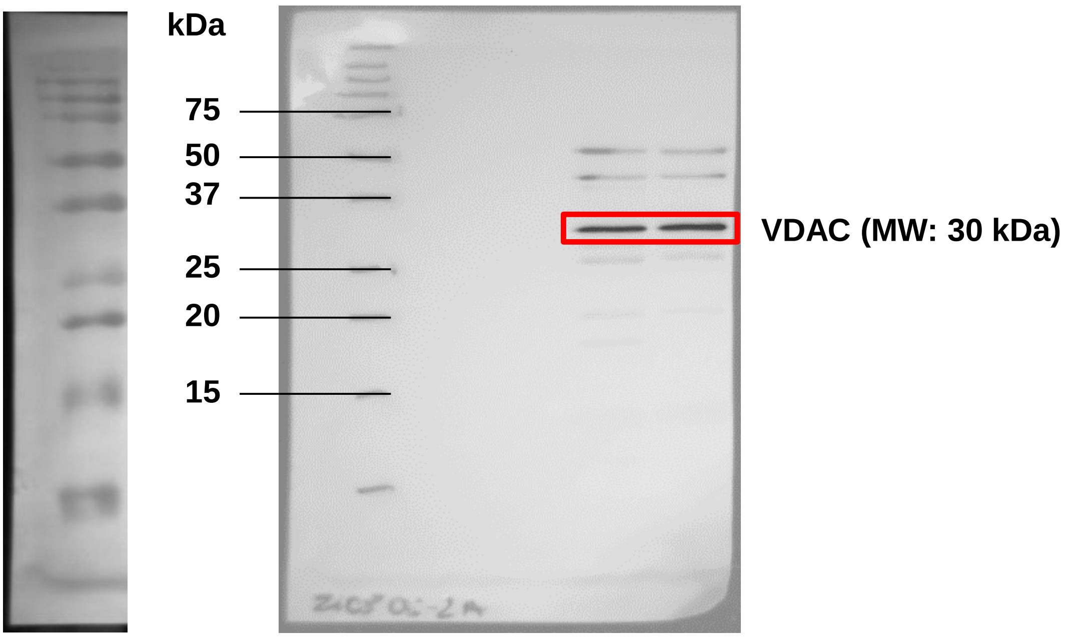
kDa
75
50
37
25
20
15
VDAC (MW: 30 kDa)

Fig. 4F

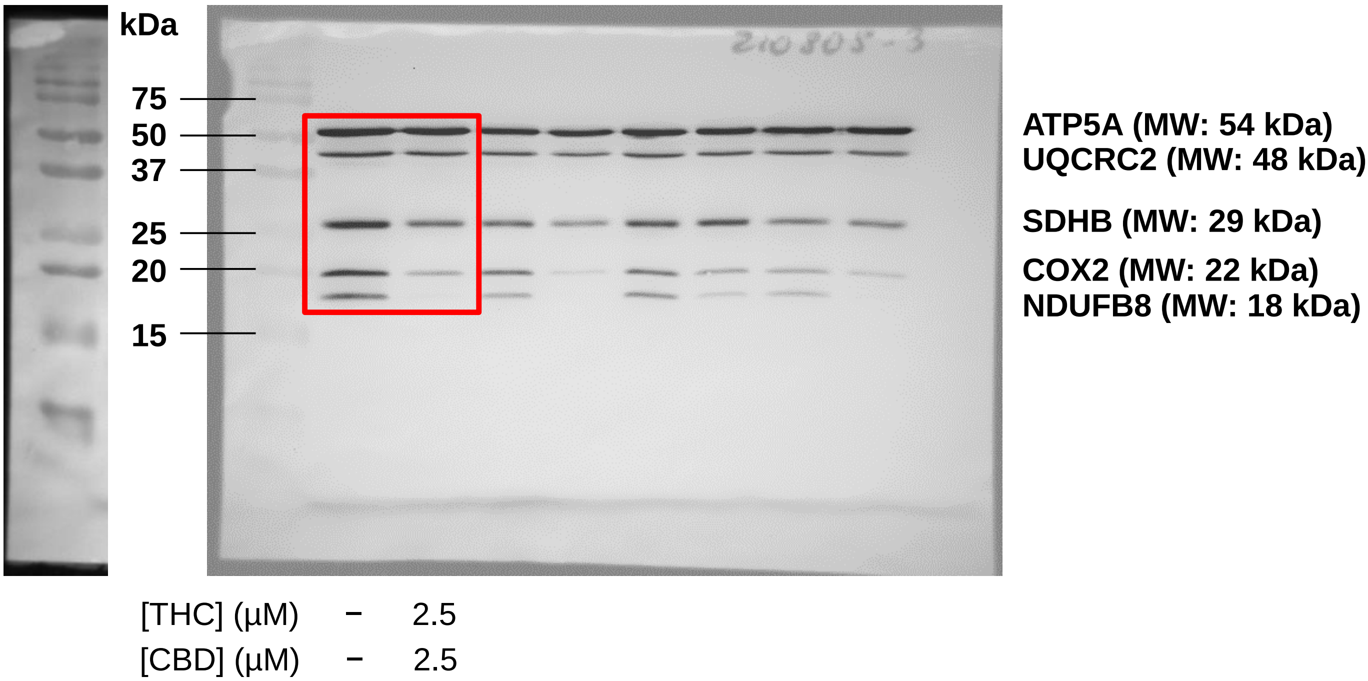
kDa
75
50
37
25
20
15
ATP5A (MW: 54 kDa)
UQCRC2 (MW: 48 kDa)
SDHB (MW: 29 kDa)
COX2 (MW: 22 kDa)
NDUFB8 (MW: 18 kDa)
[THC] (µM) – 2.5
[CBD] (µM) – 2.5

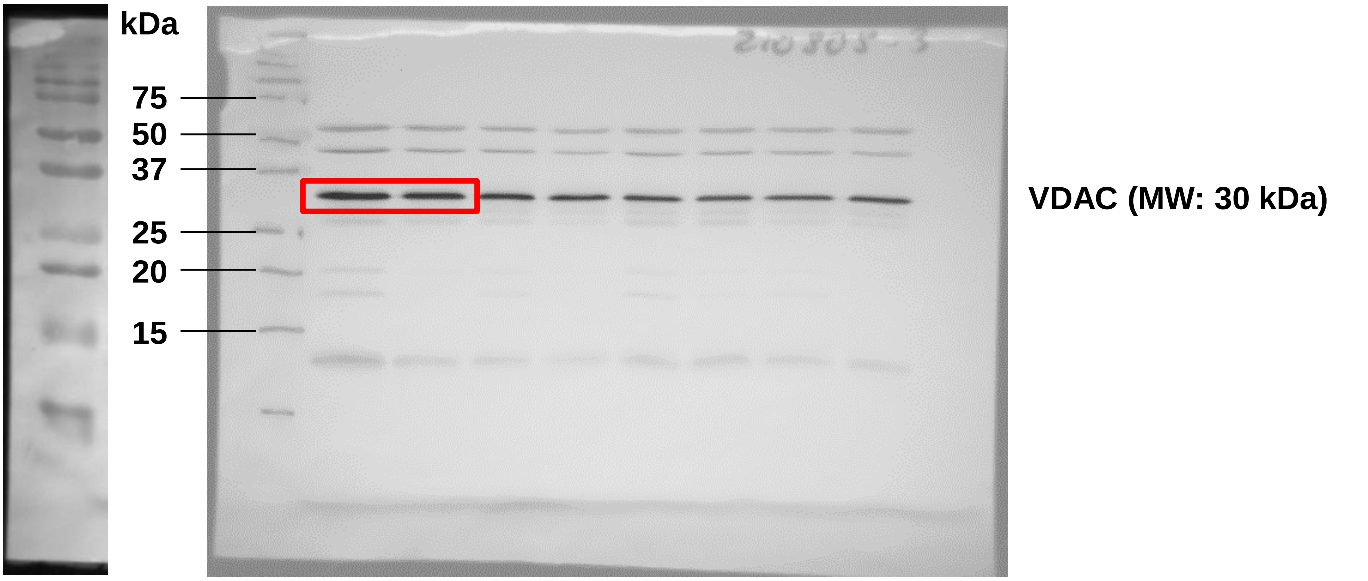
kDa
75
50
37
25
20
15
VDAC (MW: 30 kDa)

Fig. 5A

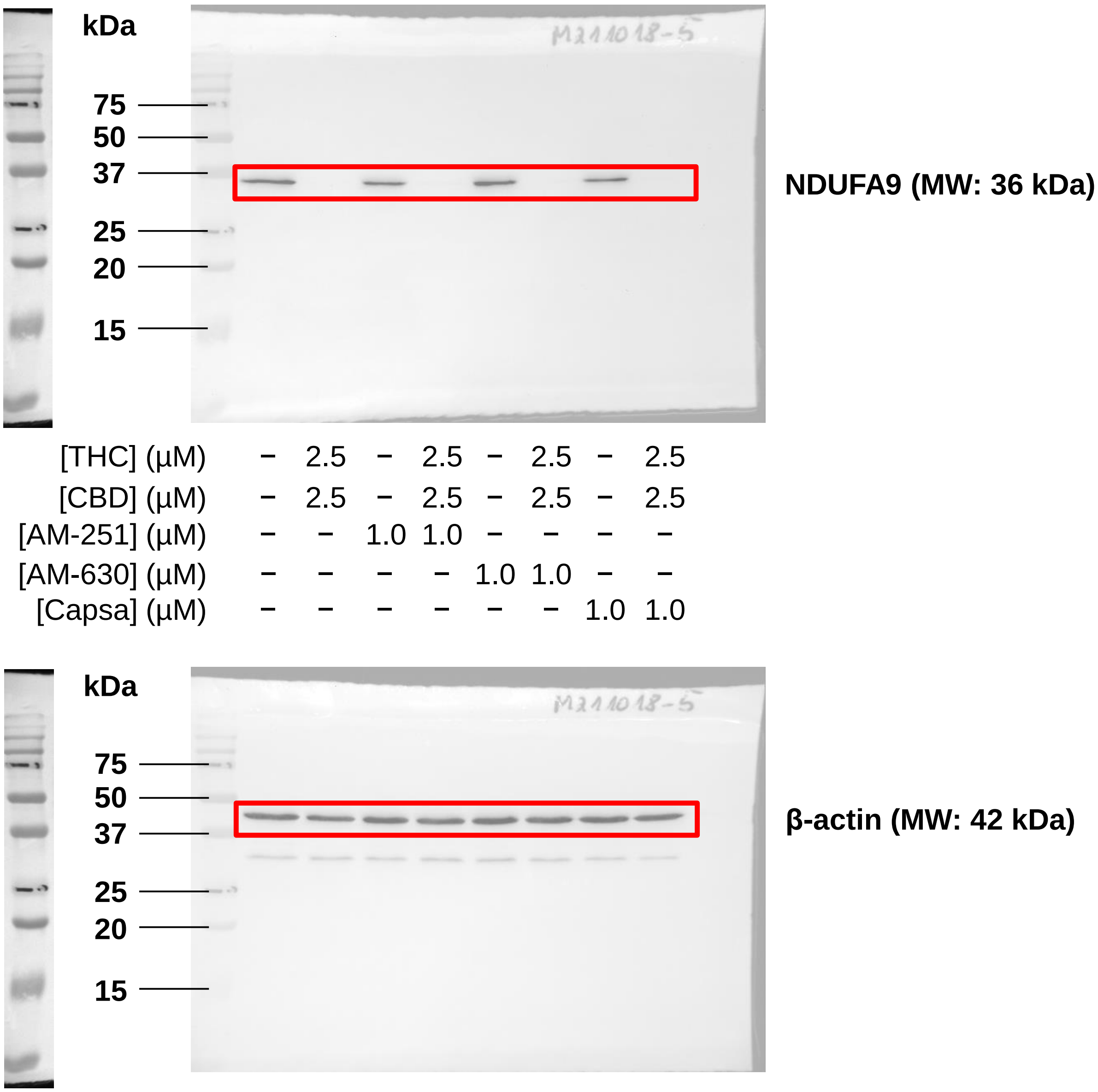
kDa
75
50
37
25
20
15
NDUFA9 (MW: 36 kDa)
[THC] (µM) – 2.5 – 2.5 – 2.5 – 2.5
[CBD] (µM) – 2.5 – 2.5 – 2.5 – 2.5
[AM-251] (µM) – – 1.0 1.0 – – – –
[AM-630] (µM) – – – – 1.0 1.0 – –
[Capsa] (µM) – – – – – – 1.0 1.0
kDa
75
50
37
25
20
15
β-actin (MW: 42 kDa)

Fig. 5B

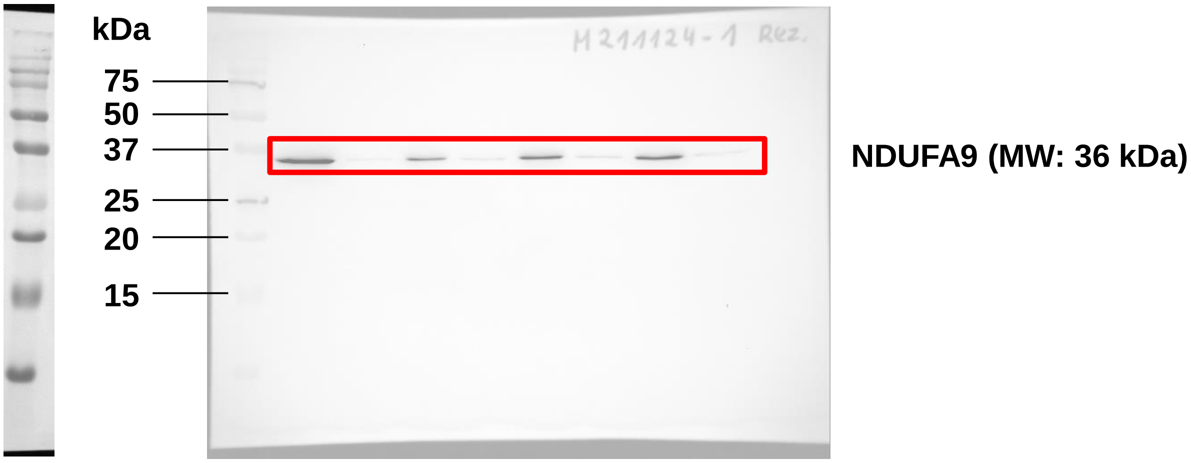

[THC] (µM)	–	2.5	–	2.5	–	2.5	–	2.5
[CBD] (µM)	–	2.5	–	2.5	–	2.5	–	2.5
[AM-251] (µM)	–	–	1.0	1.0	–	–	–	–
[AM-630] (µM)	–	–	–	–	1.0	1.0	–	–
[Capsa] (µM)	–	–	–	–	–	–	1.0	1.0

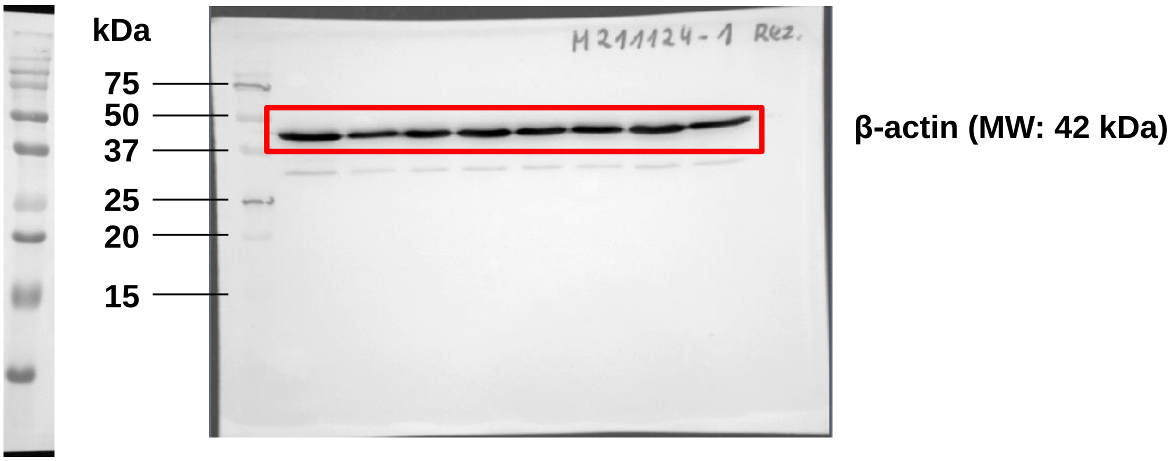

Fig. 5C

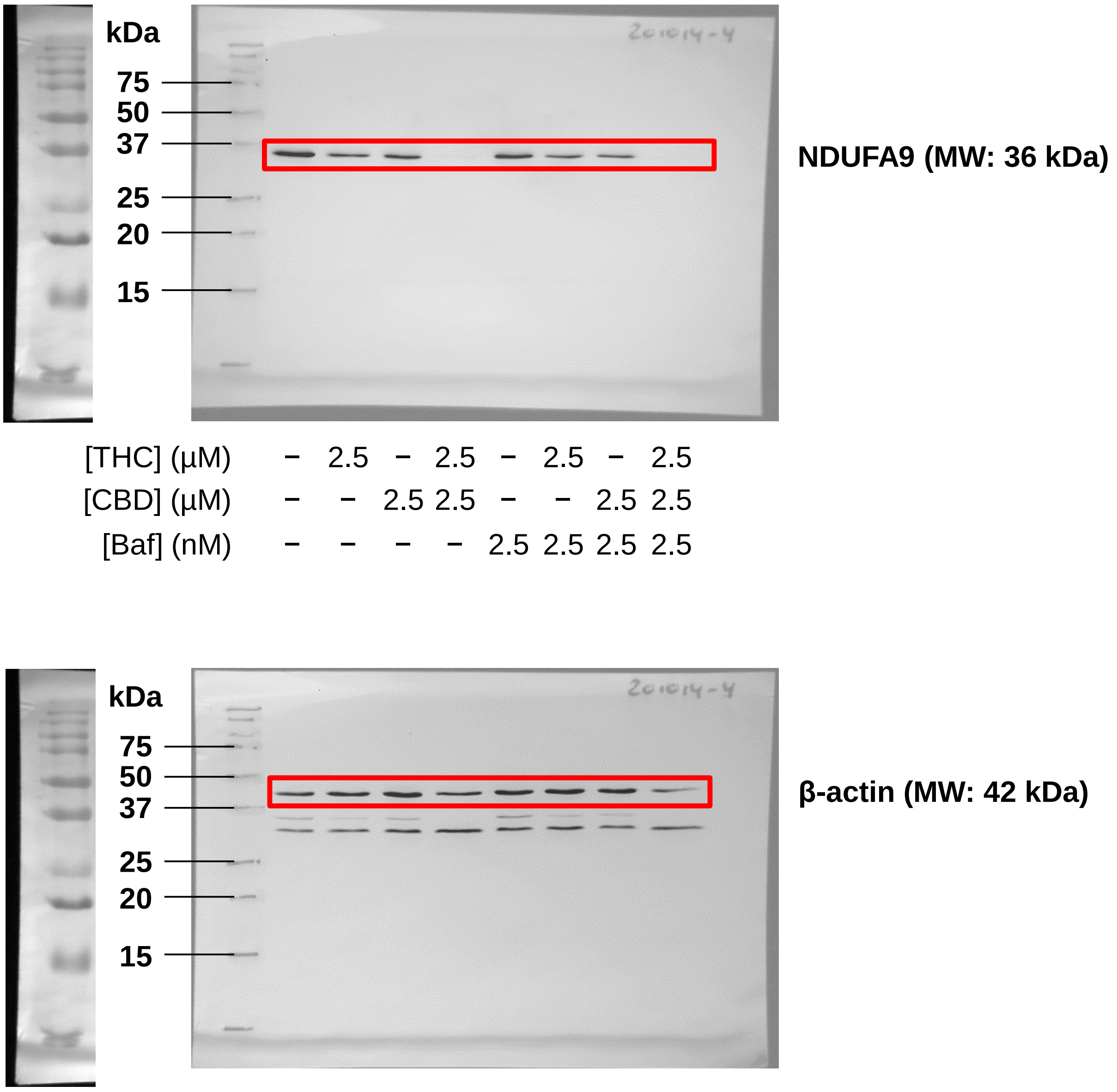
kDa
75
50
37
25
20
15
NDUFA9 (MW: 36 kDa)
[THC] (μM) – 2.5 – 2.5 – 2.5 – 2.5
[CBD] (μM) – – 2.5 2.5 – – 2.5 2.5
[Baf] (nM) – – – – 2.5 2.5 2.5 2.5
kDa
75
50
37
25
20
15
β-actin (MW: 42 kDa)

Fig. 5D

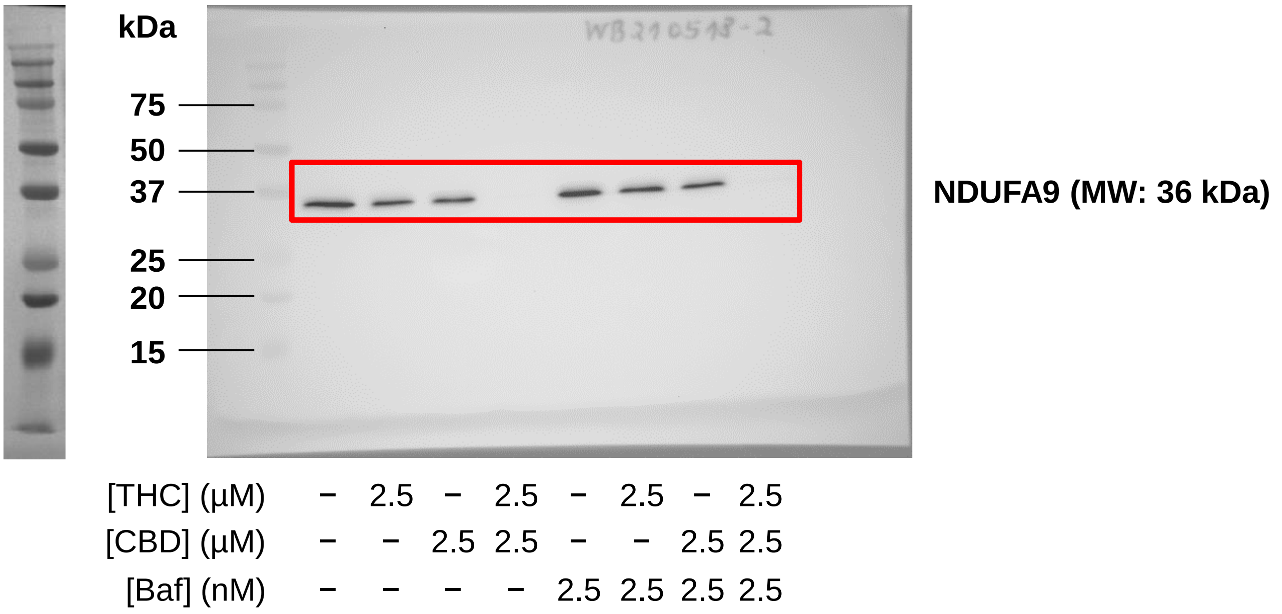

kDa
75
50
37
25
20
15
WB210518-2
NDUFA9 (MW: 36 kDa)
[THC] (μM) – 2.5 – 2.5 – 2.5 – 2.5
[CBD] (μM) – – 2.5 2.5 – – 2.5 2.5
[Baf] (nM) – – – – 2.5 2.5 2.5 2.5

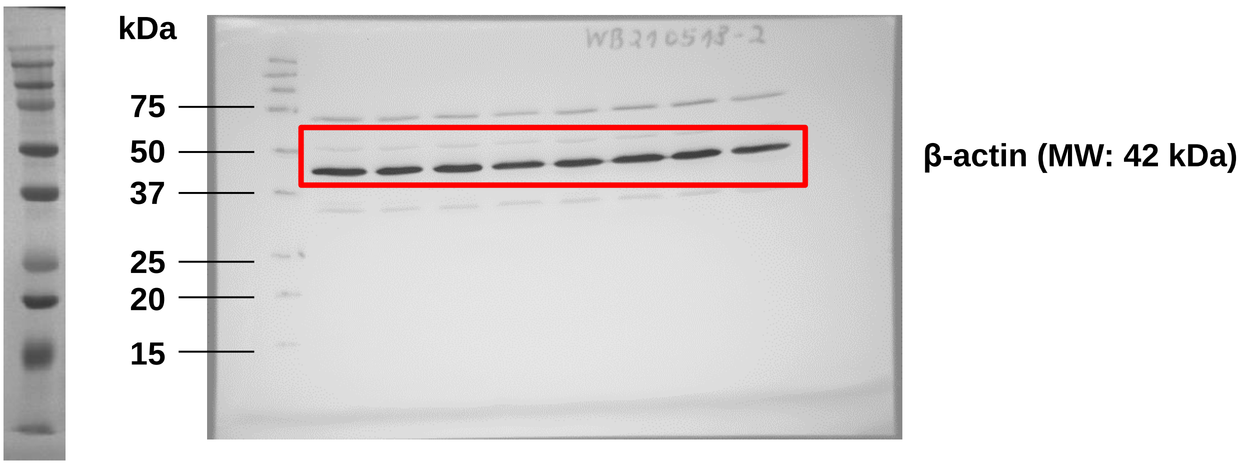

kDa
75
50
37
25
20
15
WB210518-2
β-actin (MW: 42 kDa)

Fig. 5E

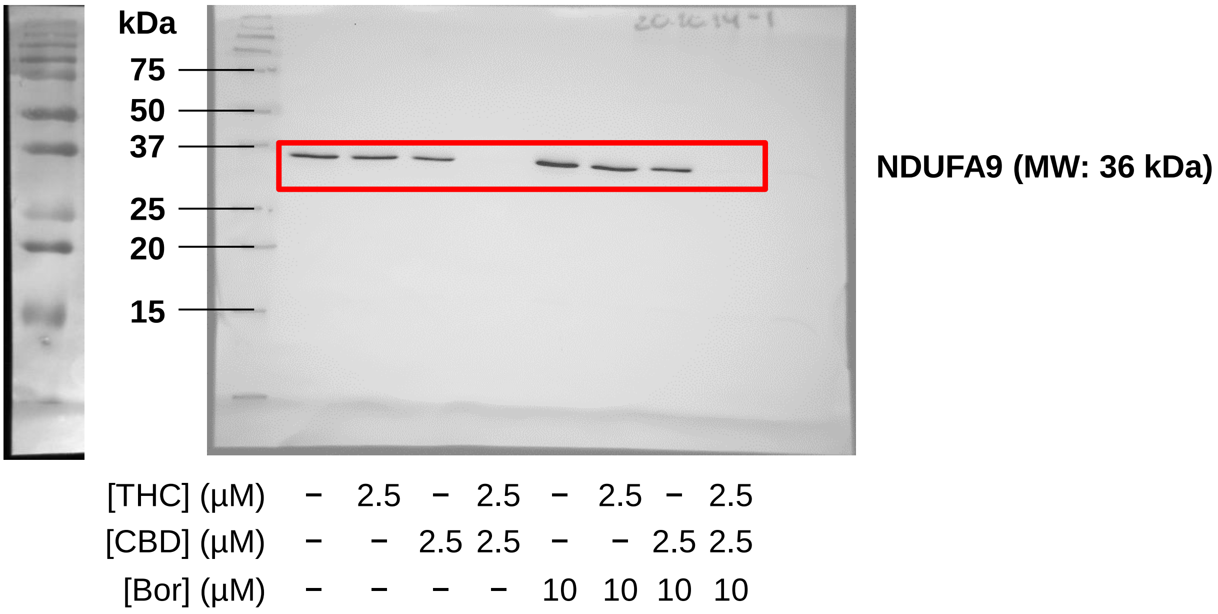
kDa
75
50
37
25
20
15
NDUFA9 (MW: 36 kDa)
[THC] (µM) – 2.5 – 2.5 – 2.5 – 2.5
[CBD] (µM) – – 2.5 2.5 – – 2.5 2.5
[Bor] (µM) – – – – 10 10 10 10

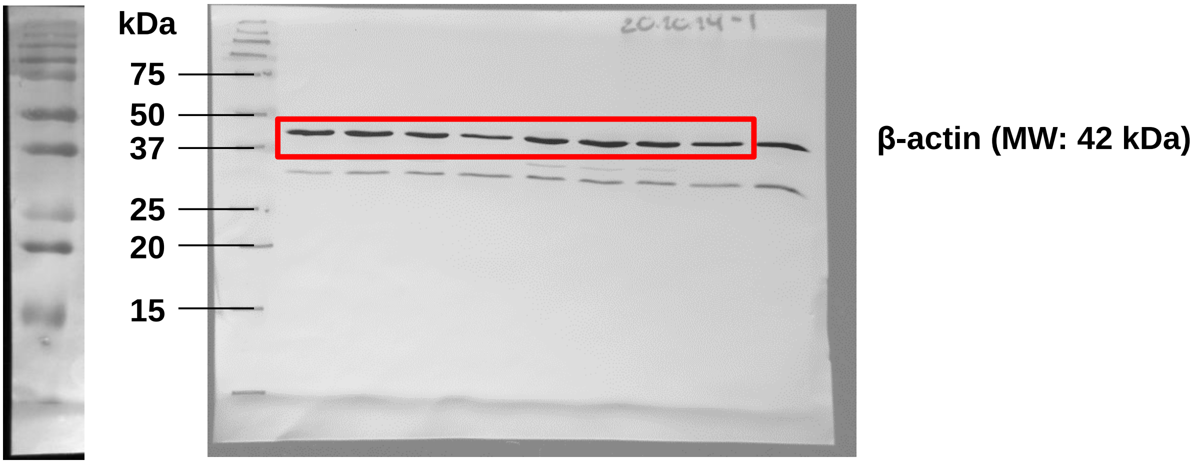
kDa
75
50
37
25
20
15
β-actin (MW: 42 kDa)

Fig. 5F

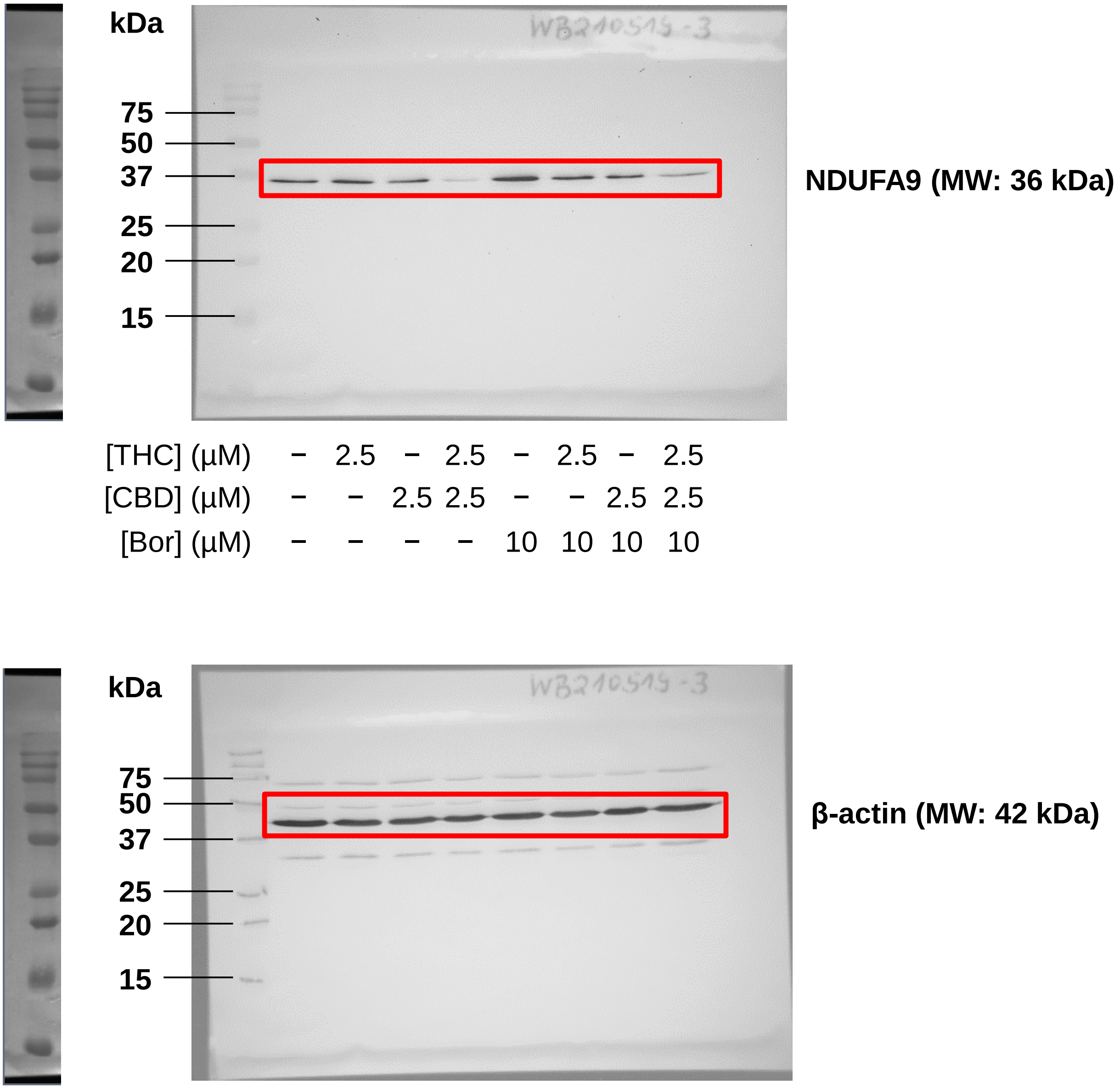
kDa
75
50
37
25
20
15
NDUFA9 (MW: 36 kDa)
[THC] (µM) – 2.5 – 2.5 – 2.5 – 2.5
[CBD] (µM) – – 2.5 2.5 – – 2.5 2.5
[Bor] (µM) – – – – 10 10 10 10
kDa
75
50
37
25
20
15
β-actin (MW: 42 kDa)

Fig. 6C

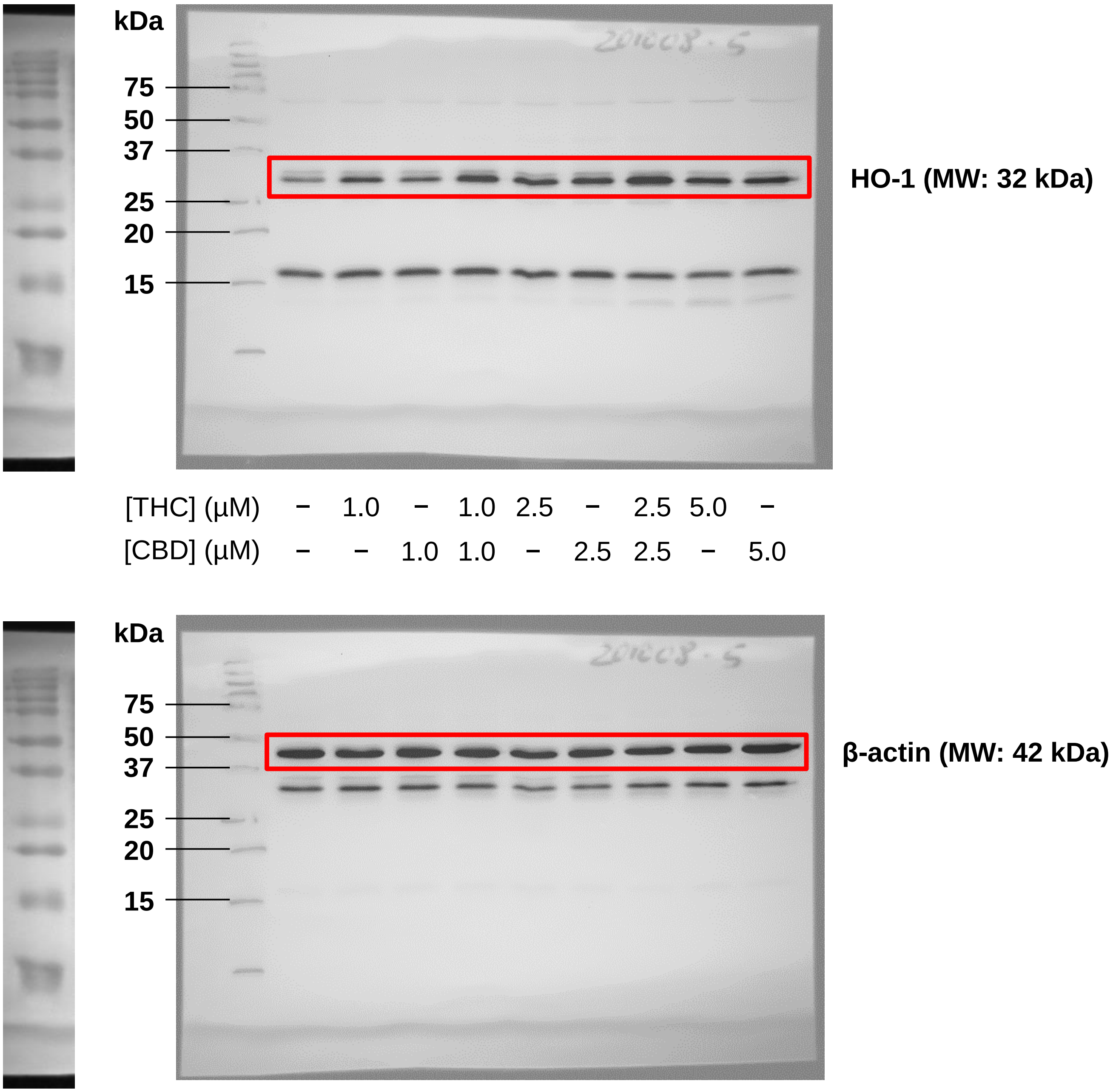

kDa
75
50
37
25
20
15
HO-1 (MW: 32 kDa)
[THC] (µM) − 1.0 − 1.0 2.5 − 2.5 5.0 −
[CBD] (µM) − − 1.0 1.0 − 2.5 2.5 − 5.0
kDa
75
50
37
25
20
15
β-actin (MW: 42 kDa)

Fig. 6D

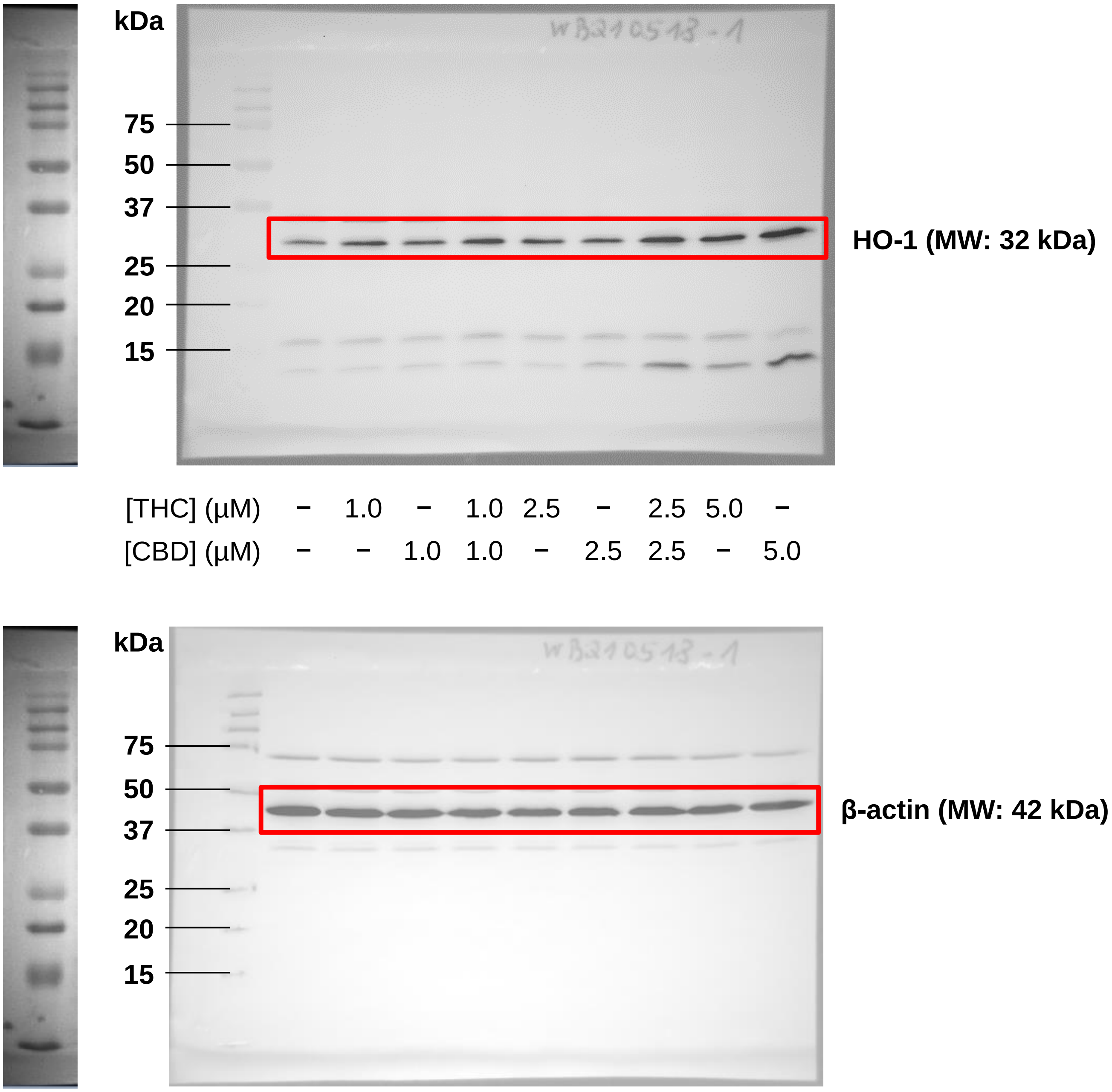
kDa
75
50
37
25
20
15
HO-1 (MW: 32 kDa)
[THC] (µM) – 1.0 – 1.0 2.5 – 2.5 5.0 –
[CBD] (µM) – – 1.0 1.0 – 2.5 2.5 – 5.0
kDa
75
50
37
25
20
15
β-actin (MW: 42 kDa)

Fig. 6E

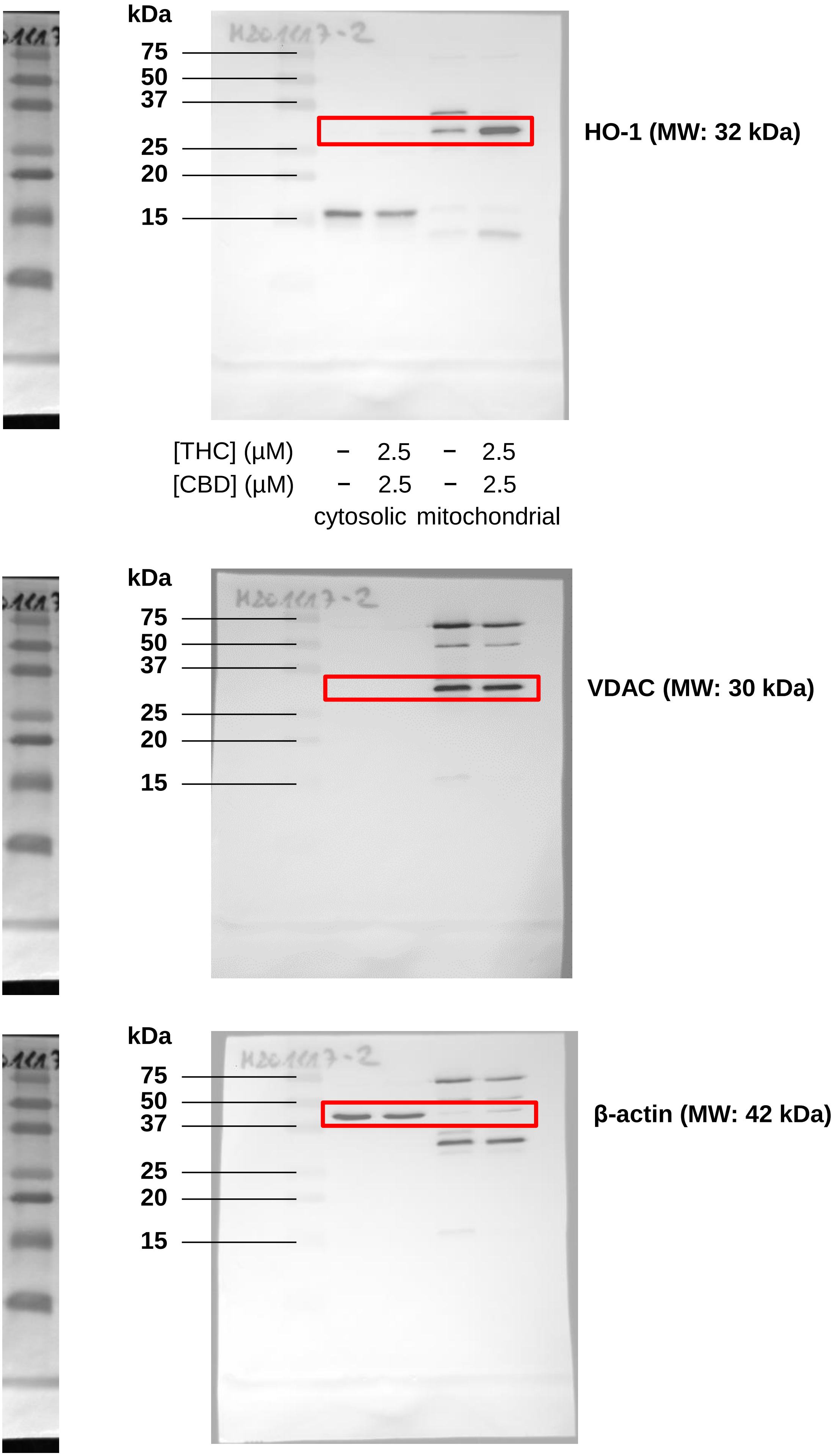
kDa
75
50
37
25
20
15
HO-1 (MW: 32 kDa)
[THC] (µM) − 2.5 − 2.5
[CBD] (µM) − 2.5 − 2.5
cytosolic mitochondrial
kDa
75
50
37
25
20
15
VDAC (MW: 30 kDa)
kDa
75
50
37
25
20
15
β-actin (MW: 42 kDa)

Fig. 6F

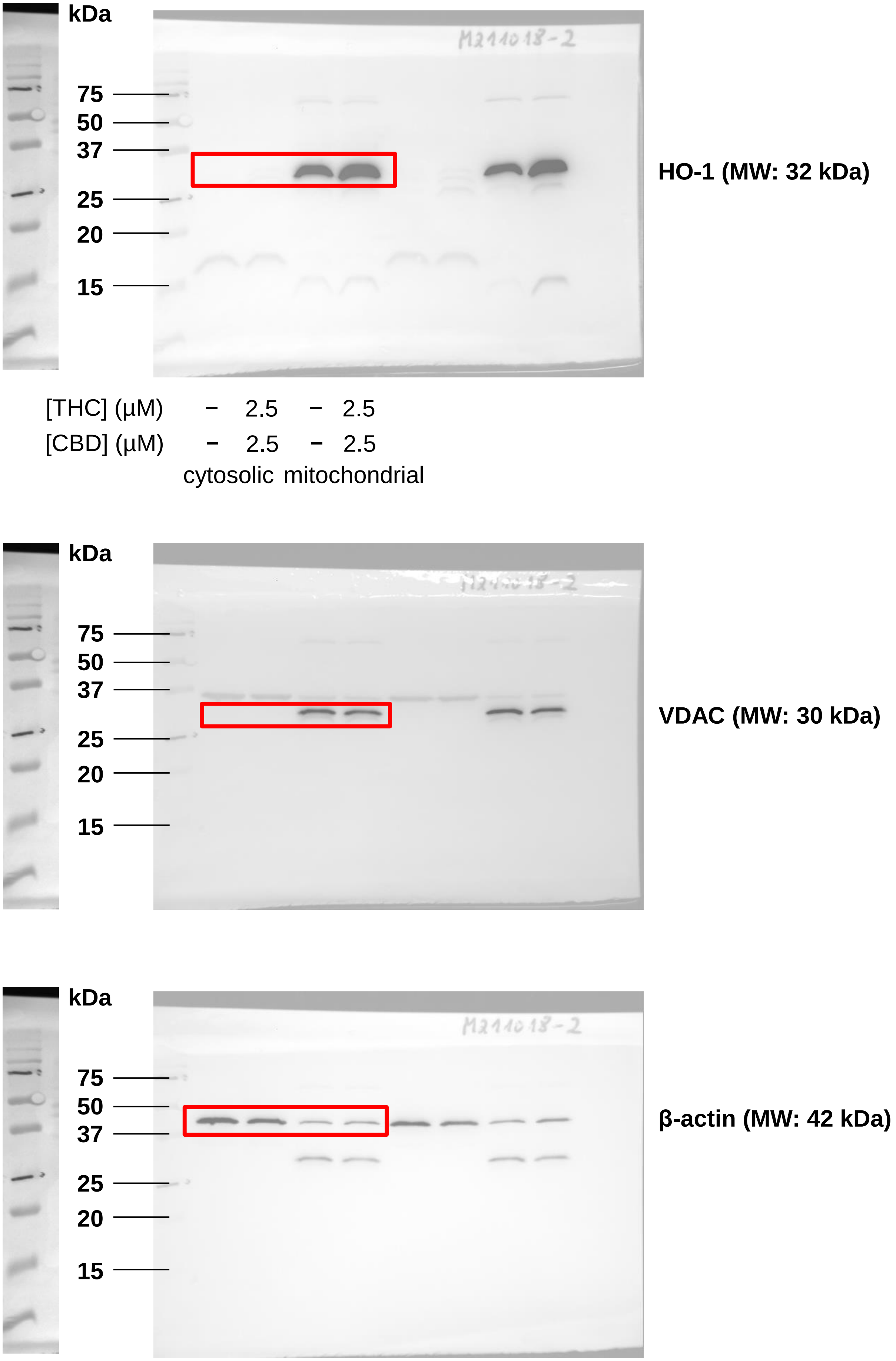
kDa
75
50
37
25
20
15
HO-1 (MW: 32 kDa)
[THC] (µM) − 2.5 − 2.5
[CBD] (µM) − 2.5 − 2.5
cytosolic mitochondrial
kDa
75
50
37
25
20
15
VDAC (MW: 30 kDa)
kDa
75
50
37
25
20
15
β-actin (MW: 42 kDa)

Fig. 6G

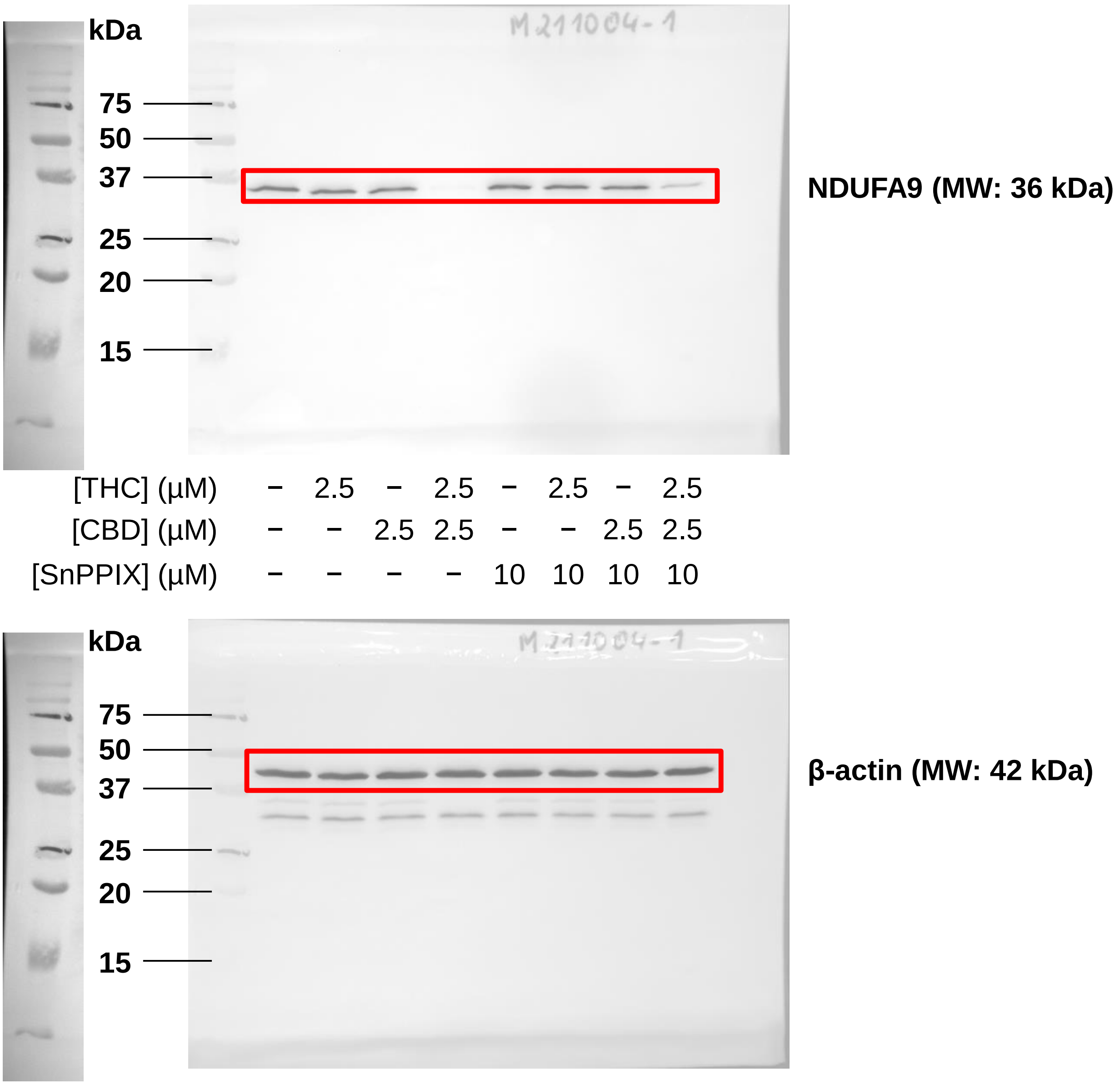
kDa
75
50
37
25
20
15
M211004-1
NDUFA9 (MW: 36 kDa)
[THC] (µM) – 2.5 – 2.5 – 2.5 – 2.5
[CBD] (µM) – – 2.5 2.5 – – 2.5 2.5
[SnPPIX] (µM) – – – – 10 10 10 10
kDa
75
50
37
25
20
15
β-actin (MW: 42 kDa)

Fig. 6H

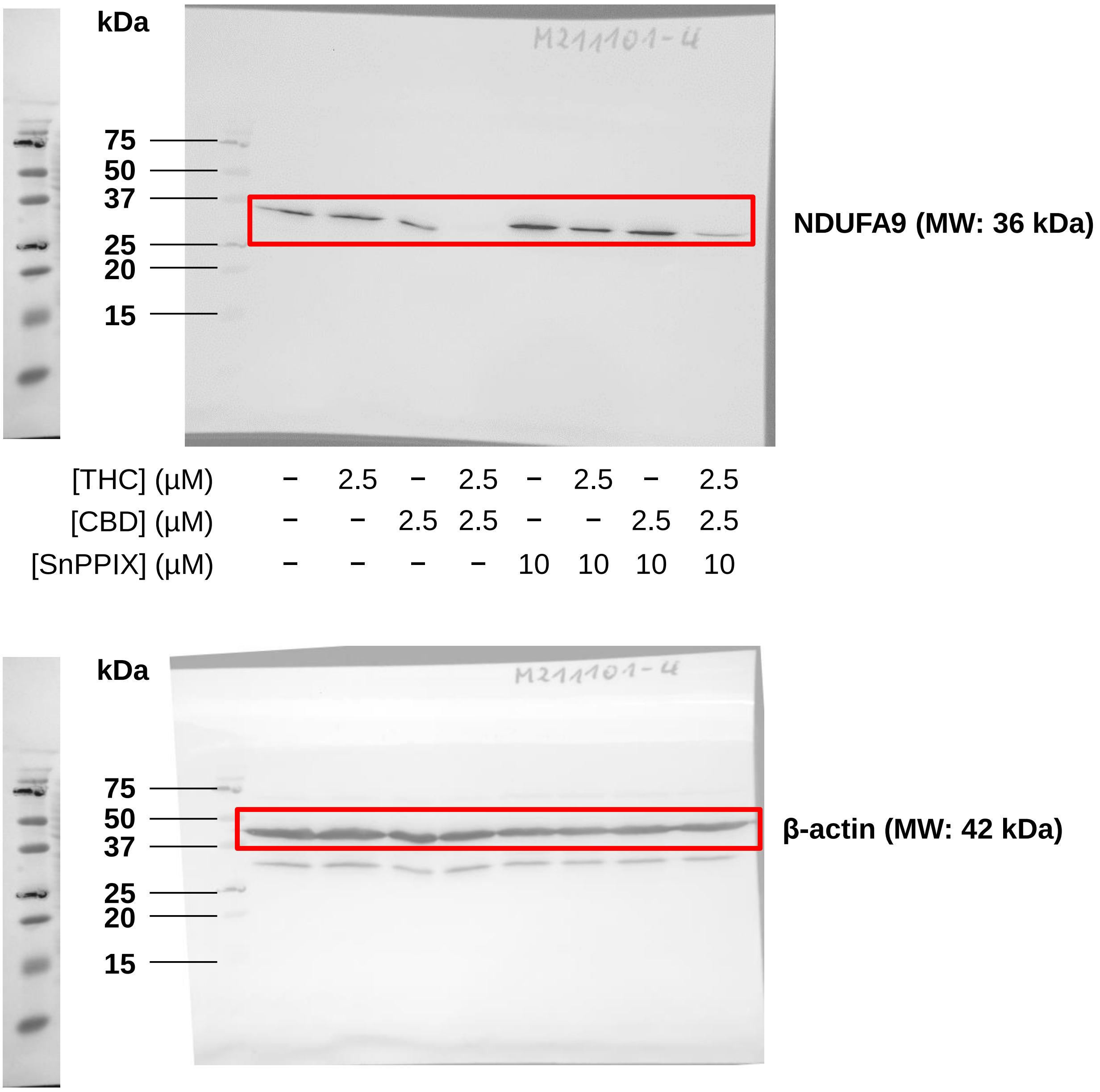
kDa
75
50
37
25
20
15
M211101-4
NDUFA9 (MW: 36 kDa)
[THC] (µM) – 2.5 – 2.5 – 2.5 – 2.5
[CBD] (µM) – – 2.5 2.5 – – 2.5 2.5
[SnPPIX] (µM) – – – – 10 10 10 10
kDa
75
50
37
25
20
15
M211101-4
β-actin (MW: 42 kDa)

Fig. S2

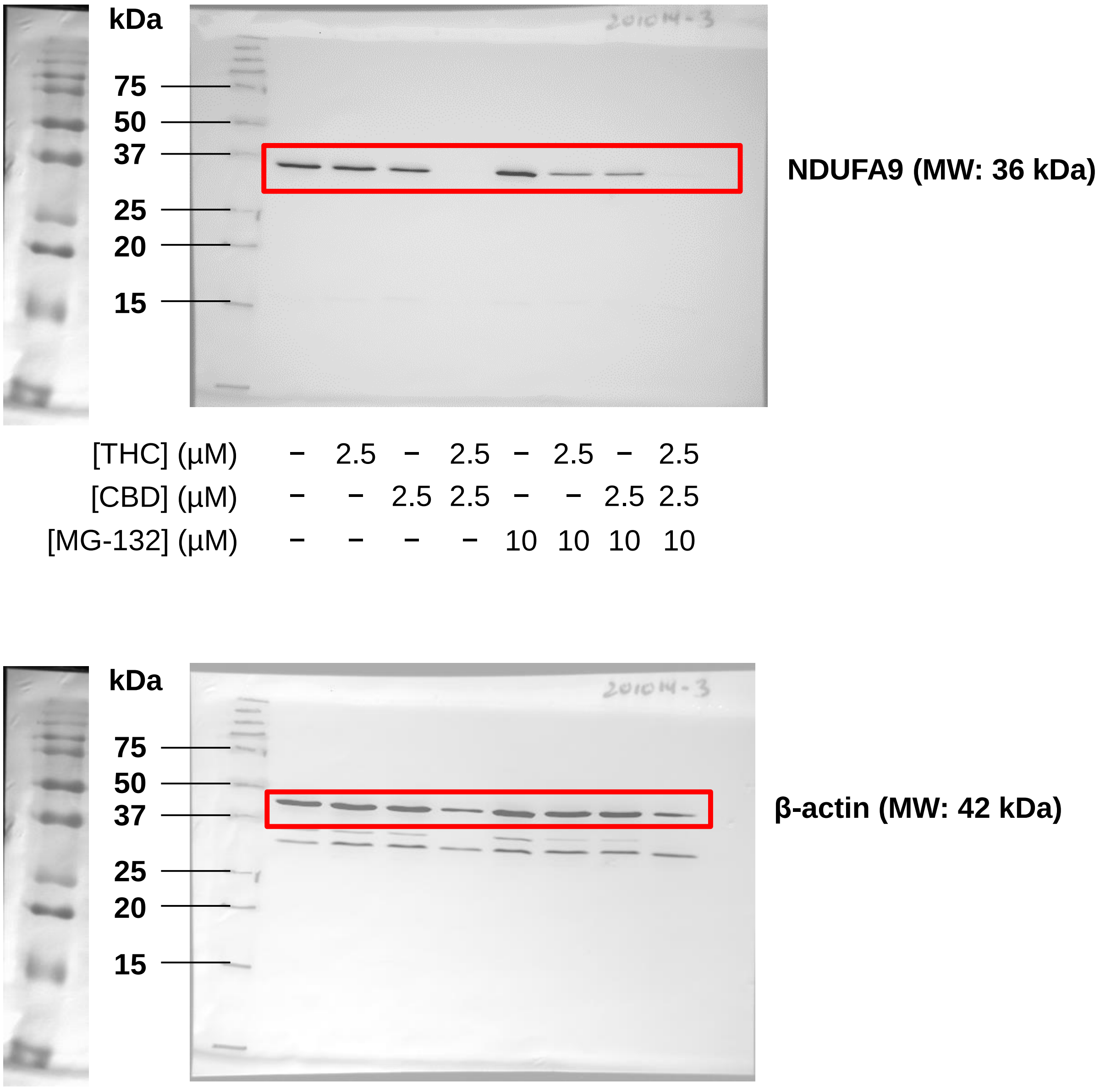
kDa
75
50
37
25
20
15
NDUFA9 (MW: 36 kDa)
[THC] (µM) – 2.5 – 2.5 – 2.5 – 2.5
[CBD] (µM) – – 2.5 2.5 – – 2.5 2.5
[MG-132] (µM) – – – – 10 10 10 10
kDa
75
50
37
25
20
15
β-actin (MW: 42 kDa)

Fig. S3A

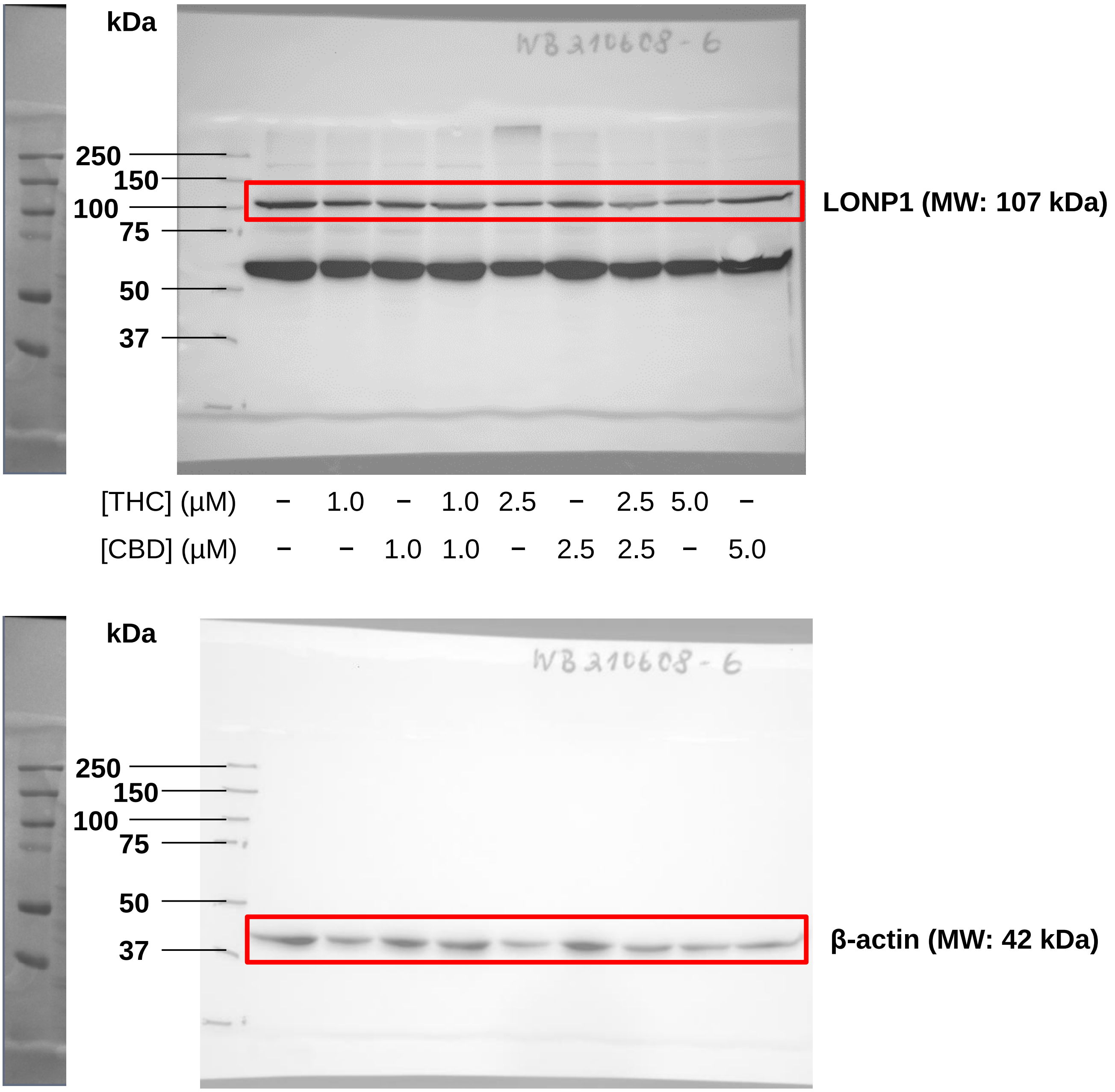

kDa
WB 210608-6
250
150
100
75
50
37
LONP1 (MW: 107 kDa)
[THC] (µM) – 1.0 – 1.0 2.5 – 2.5 5.0 –
[CBD] (µM) – – 1.0 1.0 – 2.5 2.5 – 5.0
kDa
WB 210608-6
250
150
100
75
50
37
β-actin (MW: 42 kDa)

Fig. S3B

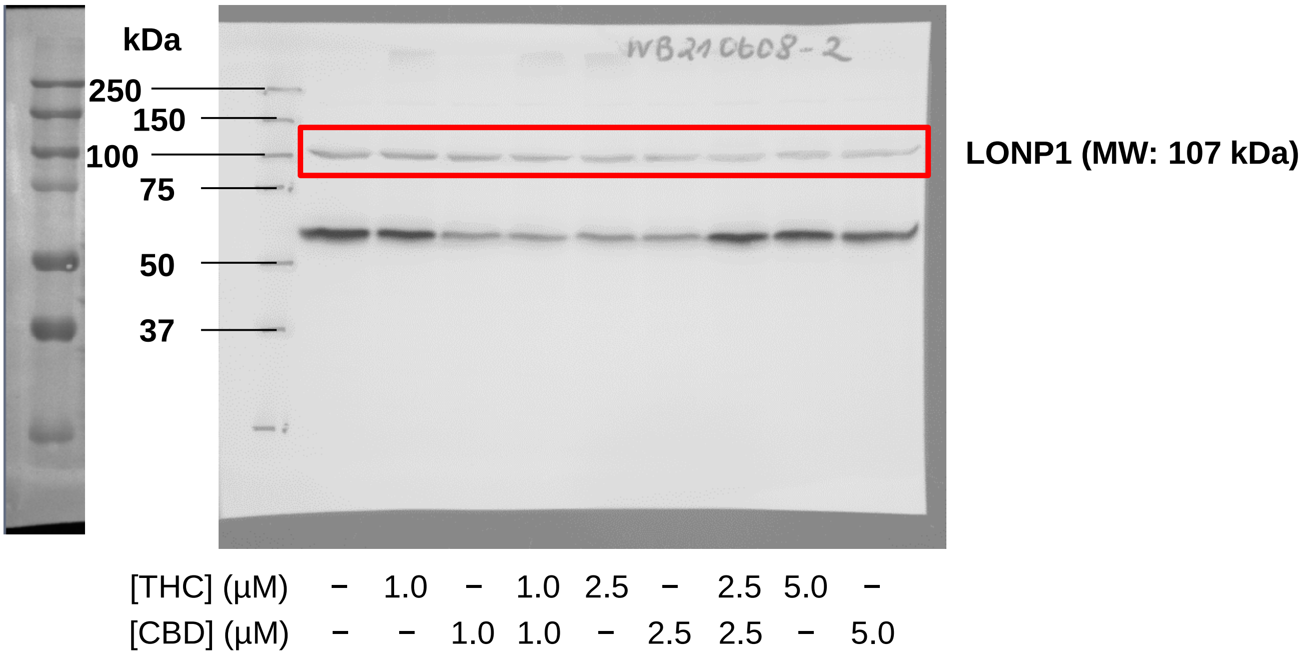
kDa
250
150
100
75
50
37
LONP1 (MW: 107 kDa)
[THC] (µM) – 1.0 – 1.0 2.5 – 2.5 5.0 –
[CBD] (µM) – – 1.0 1.0 – 2.5 2.5 – 5.0

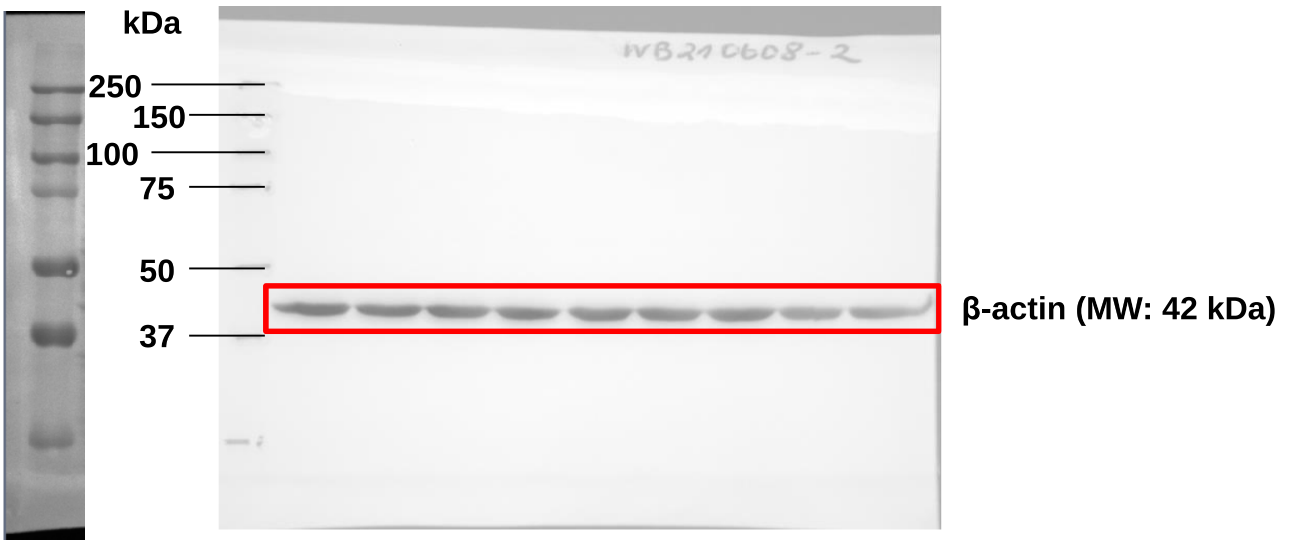
kDa
250
150
100
75
50
37
β-actin (MW: 42 kDa)
