# Supplementary material for: The Combination of Δ9-Tetrahydrocannabinol and Cannabidiol Suppresses Mitochondrial Respiration of Human Glioblastoma Cells via Downregulation of Specific Respiratory Chain Proteins
Source: Cancers (Basel). 2022 Jun 27;14(13):3129. doi: 10.3390/cancers14133129 (PMC9265124; doi:10.3390/cancers14133129)
Supplement: Supplementary file 1 [file cancers-14-03129-s001.zip › cancers-1713414-Figures S1-S3.pdf]

cancers

MDPI

Supplementary Materials

The Combination of Δ⁹-Tetrahydrocannabinol and Cannabidiol Suppresses Mitochondrial Respiration of Human Glioblastoma Cells via Downregulation of Specific Respiratory Chain Proteins

Anne Rupprecht, Ulrike Theisen, Franziska Wendt, Marcus Frank and Burkhard Hinz

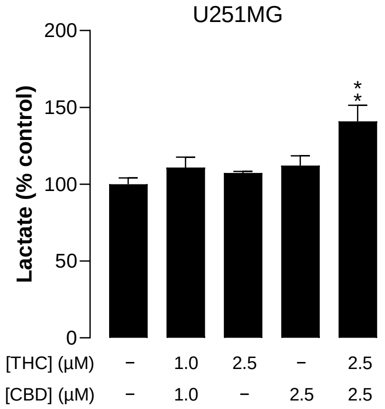

Figure S1. Influence of THC and CBD, administered alone or in combination, on lactate secretion. U251MG cells were incubated for 24 h with selected concentrations of THC and CBD or their combination (in a ratio of 1:1) as well as vehicle control. Thereafter, lactate levels were determined in supernatants. The data represent mean values ± SEM of $n = 3$ independent experiments. ** $p \leq 0.01$ vs. corresponding vehicle control, one-way ANOVA with Dunnett's post hoc test.

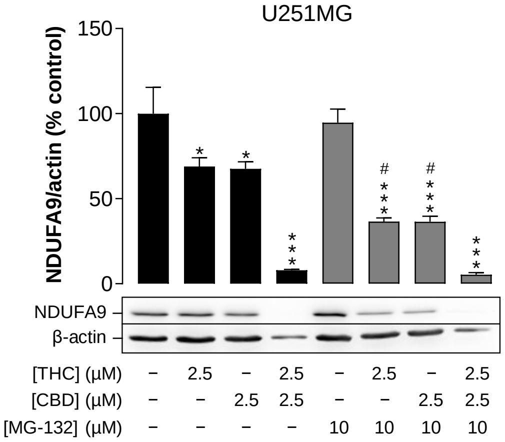

Figure S2. Role of the proteasome in downregulation of the mitochondrial subunit NDUFA9 by the combination of THC and CBD in human glioblastoma cells. U251MG were incubated with selected concentrations of THC and CBD, their combination (in a ratio of 1:1) or vehicle control for 24 h. The proteasomal inhibitor MG-132 (10 µM) was applied to the cells together with the cannabinoids and was present throughout the incubation with the cannabinoids. Subsequently, cellular total protein extracts were analysed for NDUFA9 levels by western blots. All percentage values shown refer to the vehicle control, which was set to 100%. The values shown in the bar charts are based on densitometric analyses of blots, whereby the NDUFA9 levels were normalised to β-actin. The blots shown are representative. The data are mean values ± SEM of $n = 3$ independent experiments per group. * $p \leq 0.05$, ** $p \leq 0.01$, *** $p \leq 0.001$ vs. corresponding vehicle control, # $p \leq 0.05$ vs. corresponding THC or CBD group without MG-132, one-way ANOVA with Bonferroni's post hoc test.

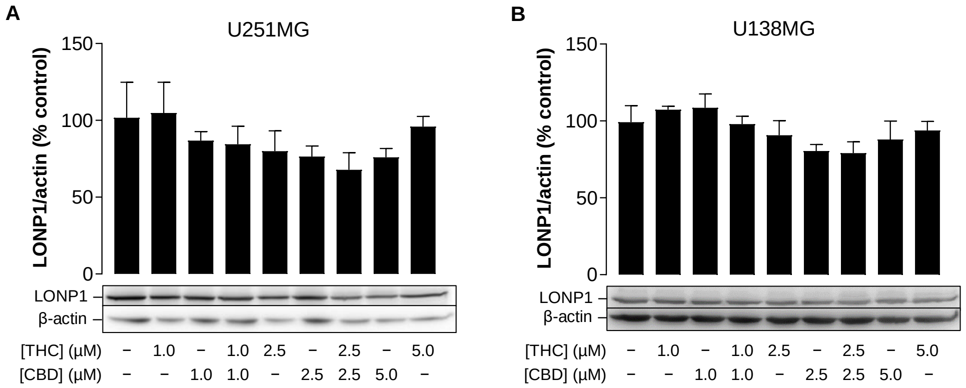

Figure S3. Influence of THC and CBD, administered alone or in combination, on LONP levels in human glioblastoma cells. U251MG and U138MG cells were incubated for 24 h with selected concentrations of THC and CBD or their combination (in a ratio of 1:1) as well as vehicle control. Subsequently, cellular total protein extracts were analysed for NDUFA9 levels by western blots. All percentage values shown refer to the vehicle control, which was set to 100%. The values shown in the bar charts are based on densitometric analyses of blots, whereby the LONP1 levels were normalised to β-actin. The blots shown are representative. The data are mean values ± SEM of n = 3–4 independent experiments per group. Statistically significant differences between the treatment groups and the corresponding vehicle control were excluded by one-way ANOVA with Dunnett's post hoc test.
